# Characterizing RNA stability genome-wide through combined analysis of PRO-seq and RNA-seq data

**DOI:** 10.1186/s12915-021-00949-x

**Published:** 2021-02-15

**Authors:** Amit Blumberg, Yixin Zhao, Yi-Fei Huang, Noah Dukler, Edward J. Rice, Alexandra G. Chivu, Katie Krumholz, Charles G. Danko, Adam Siepel

**Affiliations:** 1 Simons Center for Quantitative Biology, Cold Spring Harbor Laboratory, Cold Spring Harbor, NY USA; 2 Present Address: Department of Biology and Huck Institutes of the Life Sciences, Pennsylvania State University, University Park, PA USA; 3 Baker Institute for Animal Health, College of Veterinary Medicine, Cornell University, Ithaca, NY USA

**Keywords:** RNA half-life, RNA splicing, Epigenomics, PRO-seq, Structural equation modeling

## Abstract

**Background:**

The concentrations of distinct types of RNA in cells result from a dynamic equilibrium between RNA synthesis and decay. Despite the critical importance of RNA decay rates, current approaches for measuring them are generally labor-intensive, limited in sensitivity, and/or disruptive to normal cellular processes. Here, we introduce a simple method for estimating relative RNA half-lives that is based on two standard and widely available high-throughput assays: Precision Run-On sequencing (PRO-seq) and RNA sequencing (RNA-seq).

**Results:**

Our method treats PRO-seq as a measure of transcription rate and RNA-seq as a measure of RNA concentration, and estimates the rate of RNA decay required for a steady-state equilibrium. We show that this approach can be used to assay relative RNA half-lives genome-wide, with good accuracy and sensitivity for both coding and noncoding transcription units. Using a structural equation model (SEM), we test several features of transcription units, nearby DNA sequences, and nearby epigenomic marks for associations with RNA stability after controlling for their effects on transcription. We find that RNA splicing-related features are positively correlated with RNA stability, whereas features related to miRNA binding and DNA methylation are negatively correlated with RNA stability. Furthermore, we find that a measure based on U1 binding and polyadenylation sites distinguishes between unstable noncoding and stable coding transcripts but is not predictive of relative stability within the mRNA or lincRNA classes. We also identify several histone modifications that are associated with RNA stability.

**Conclusion:**

We introduce an approach for estimating the relative half-lives of individual RNAs. Together, our estimation method and systematic analysis shed light on the pervasive impacts of RNA stability on cellular RNA concentrations.

**Supplementary Information:**

The online version contains supplementary material available at 10.1186/s12915-021-00949-x.

## Background

Gene regulation is an exquisitely complex process that operates at all stages of gene expression, ranging from pre-transcriptional chromatin remodeling to post-translational modification of proteins. However, the concentration of RNA molecules in the cell appears to serve as the primary target of many regulatory mechanisms. Many studies of gene regulation focus on the production of RNA, often at the stages of transcriptional pre-initiation, initiation, or release from pausing into productive elongation. RNA concentrations, however, result from a dynamic equilibrium between the production of new RNA molecules and their decay [1–7]. Indeed, bulk differences in RNA concentrations across types of transcription units (TUs) often result from differences in RNA decay rates rather than differences in production rates. For example, protein-coding mRNAs, on average, are relatively stable (having low rates of decay), whereas lincRNAs are less stable, and enhancer RNAs (eRNAs) and other short noncoding RNAs tend to be extremely unstable [3, 6, 8, 9]. Among protein-coding genes, mRNAs associated with housekeeping functions tend to be stable, whereas those associated with regulation of transcription and apoptosis tend to have much shorter half-lives, probably to enable RNA concentrations to change rapidly in response to changing conditions [4, 6, 7, 10, 11]. In some cases, RNA decay is accelerated by condition- or cell type-specific expression of microRNAs or RNA-binding proteins [3, 12].

Over several decades, investigators have developed numerous methods for measuring RNA decay rates or half-lives [13–15]. A classical approach to this problem is to measure the decay in RNA abundance over time following inhibition of transcription, often using actinomycin D [1, 7, 16]. More recently, many studies have employed a strategy that is less disruptive to cellular physiology, based on metabolic labeling of RNA transcripts with modified nucleotides. In this approach, the relative proportions of labeled and unlabeled transcripts are quantified as they change over time, following an initial introduction or removal of labeled nucleotides [6, 15]. Today, metabolic labeling is most commonly accomplished using the nucleotide analog 4-thiouridine (4sU), which is rapidly taken up by animal cells and can be biotinylated for affinity purification [2, 3, 8, 17–19]. Related methods use chemical conversion of 4sU nucleotide analogs to allow identification by sequencing and avoid the need for affinity purification [10, 20]. In most of these assays, sample preparation and sequencing must be performed in a time course, making the protocols labor-intensive and dependent on the availability of abundant and homogeneous sample material (typically a cell culture). Many of these methods also have limited sensitivity for low-abundance transcripts. Owing to a variety of limitations, estimates of RNA half-lives tend to vary considerably across assays, with median half-lives often differing by factors of 2–3 or more [6, 15]. As yet, there exists no general-purpose assay for RNA half-life that is as robust, sensitive, or versatile as RNA-seq [12, 21, 22] is for measuring cellular RNA concentrations, or PRO-seq [23] and NET-seq [24] are for mapping engaged RNA polymerases.

Recently, it has been shown that changes to RNA half-lives can be identified in a simpler manner, by working directly from high-throughput RNA-seq data [12, 21, 22, 25]. The essential idea behind these methods is to treat RNA-seq read counts obtained from introns as a surrogate for transcription rates, and read counts obtained from exons as a surrogate for RNA abundance. Changes in half-life are then inferred from changes to the ratio of these quantities, under the assumption of a steady-state equilibrium between RNA production and decay. This approach assumes intronic read counts are representative of pre-mRNA abundances, when in fact they may derive from a variety of sources, and it can require a correction for differences in RNA processing rates [21]. Moreover, the dependency on intronic reads limits the method to intron-containing transcription units that are transcribed at relatively high levels. Nevertheless, this simple approach requires no time course, metabolic labeling, transcriptional inhibition, or indeed any experimental innovation beyond standard RNA-seq, making it an inexpensive and effective strategy for identifying genes undergoing cell type- or condition-specific decay [12, 21, 22].

In this article, we show that this same general approach—but using a measure of nascent transcription based on PRO-seq rather than intronic RNA-seq reads—results in improved estimates of relative RNA half-life. Our approach requires only two standard and widely applicable experimental protocols—PRO-seq and RNA-seq. It applies to intron-less as well as intron-containing transcription units, it requires no correction for RNA-processing rates, it makes efficient use of the available sample material and can be extended to tissue samples using ChRO-seq [26], it is relatively nondisruptive to the biological processes under study, and it is sufficiently sensitive to assay TUs expressed at low levels, including many noncoding RNAs (see Additional file 1: Table S1 for a summary of advantages [26, 27]). We show, through a series of analyses, that these combined RNA-seq and PRO-seq measurements are a powerful means for assaying RNA stability that can reveal possible determinants of RNA decay.

## Results

### Matched PRO-seq and RNA-seq measurements are generally well correlated but suggest reduced stability of noncoding RNAs

We first compared PRO-seq and RNA-seq measurements for various TUs from across the human genome, to assess the degree to which transcriptional activity, as assayed by PRO-seq, is predictive of steady-state RNA concentrations, as assayed by RNA-seq. We obtained previously published PRO-seq [28] and rRNA-depleted poly-A+ RNA-seq data for K562 cells (see the “Methods” section), and pooled the two replicates available for each data type after verifying high concordance between them (Additional file 2: Figure S1). We also collected new PRO-seq and total-RNA RNA-seq data for K562 cells and obtained similar results (see the “Methods” section), but we focus here on the previously published data, which exhibited somewhat reduced technical and biological noise (see the “Discussion” section). When analyzing these data, we considered all annotated TUs in GENCODE [29], dividing them into mRNA (*n* = 11,011), lincRNA (*n* = 1143), antisense (*n* = 1066), and pseudogene (*n* = 590) classes. We quantified expression by the total number of mapped reads in transcripts per million (TPM), a measure that normalizes by both library size and TU length, and discarded TUs with insufficient read counts from either assay. Notably, we excluded the first 500 bp downstream of the TSS and 500 bp upstream of TES for PRO-seq to avoid a bias from promoter-proximal pausing and polymerase deceleration [23] (see the “Methods” section).

**Fig. 1:**
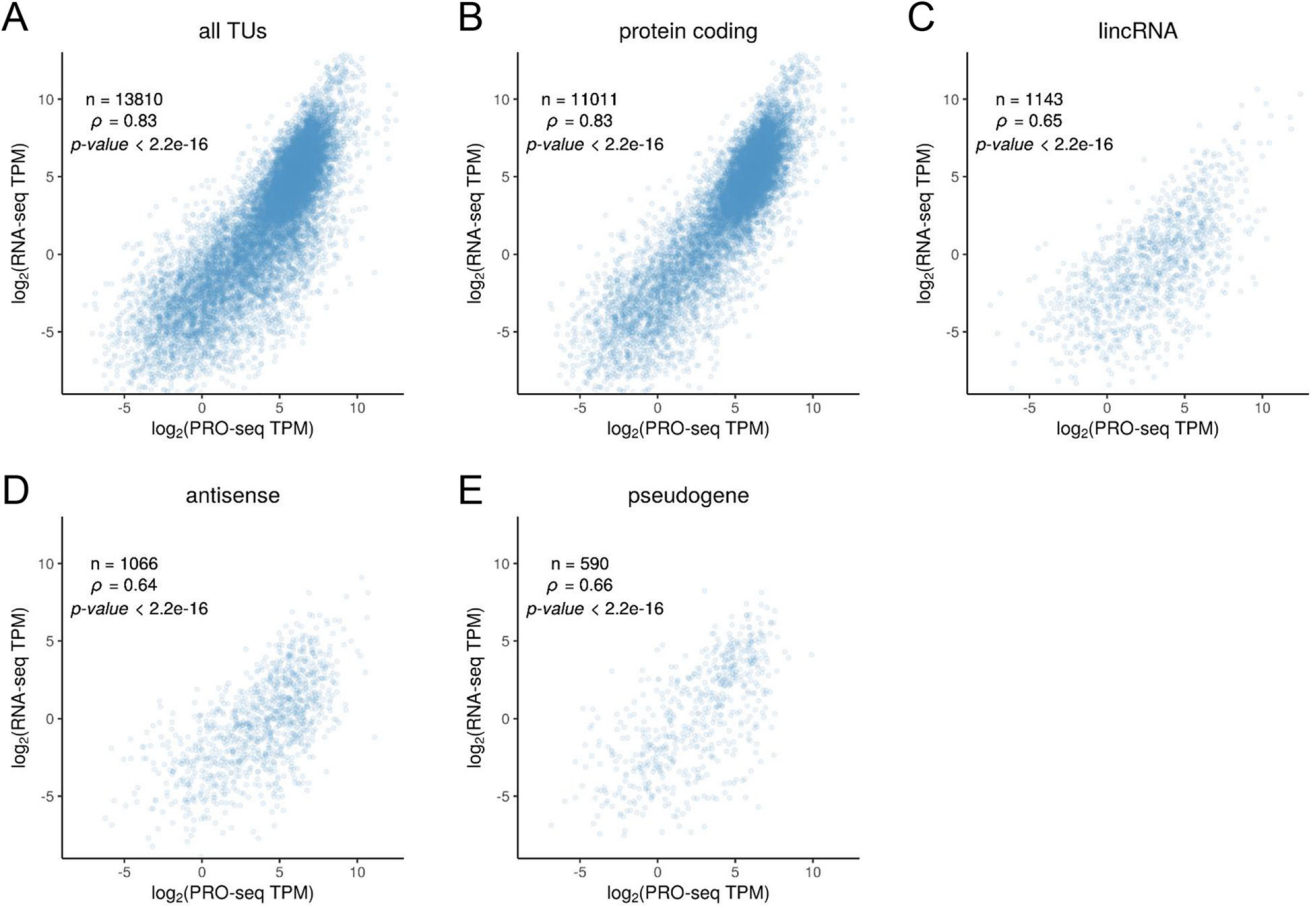
Scatter plots of PRO-seq vs. RNA-seq read counts for transcription units (TUs) in K562 cells, both shown in units of log_2_ transcripts per million (TPM) (see the “Methods” section). Panels describe **a** all annotated TUs (*n* = 13,810), **b** protein-coding mRNAs (*n* = 11,011), **c** intergenic lincRNAs (*n* = 1143), **d** intragenic antisense noncoding genes (*n* = 1066), and **e** pseudogenes (*n* = 590), all from GENCODE [29]. For each plot, the Spearman’s rank correlation coefficient (*ρ*) is shown. TUs with values of zero along either axis have been omitted. Notice that as one proceeds from **b** to **e**, from mRNAs to noncoding RNAs and pseudogenes, there is a general decrease in *ρ*, indicating greater variability of steady-state RNA concentrations at each transcription level

We found that the PRO-seq and RNA-seq measurements were well correlated overall, with Spearman’s *ρ* = 0.83 (Fig. 1a), suggesting that transcription explains the majority of the variance in mRNA levels. A parallel analysis based on pooled intronic reads from the same RNA-seq libraries showed only a slightly higher correlation, with *ρ* = 0.85 (Additional file 2: Figure S2). At the same time, there were considerable differences in the degree of correlation across classes of TUs, ranging from a high of *ρ* = 0.83 for protein-coding mRNAs to *ρ* = 0.65 for lincRNAs, *ρ* = 0.64 for antisense genes, and *ρ* = 0.66 for pseudogenes (Fig. 1b–e). We observed similar patterns for both intron-containing and intron-less genes (Additional file 2: Figures S3 & S4). Together, these observations suggest that RNA decay has a more pronounced effect on steady-state RNA levels in noncoding RNAs and pseudogenes. These differences remain when TUs are matched by expression level (see the “Methods” section; Additional file 2: Figure S5), when our own K562 data is used (Additional file 2: Figure S6), and when the HeLa cells are evaluated instead (Additional file 2: Figure S7).

Elongation rate is an important potential confounding factor in this analysis, because the PRO-seq density does not directly reflect the synthesis rate of RNA, but rather the synthesis rate divided by the elongation rate, which is known to vary across TUs [30]. However, when we correct for elongation rate using published estimates for K562 cells [31], we find that the correlation with RNA-seq measurements does not improve, and indeed, declines slightly (Additional file 2: Figure S8). Thus, the observed relationships between PRO-seq and RNA-seq measurements do not appear to be driven primarily by differences in elongation rate (see the “Methods” and “Discussion” sections).

### Relative RNA half-life can be estimated from the RNA-seq/PRO-seq ratio

**Fig. 2:**
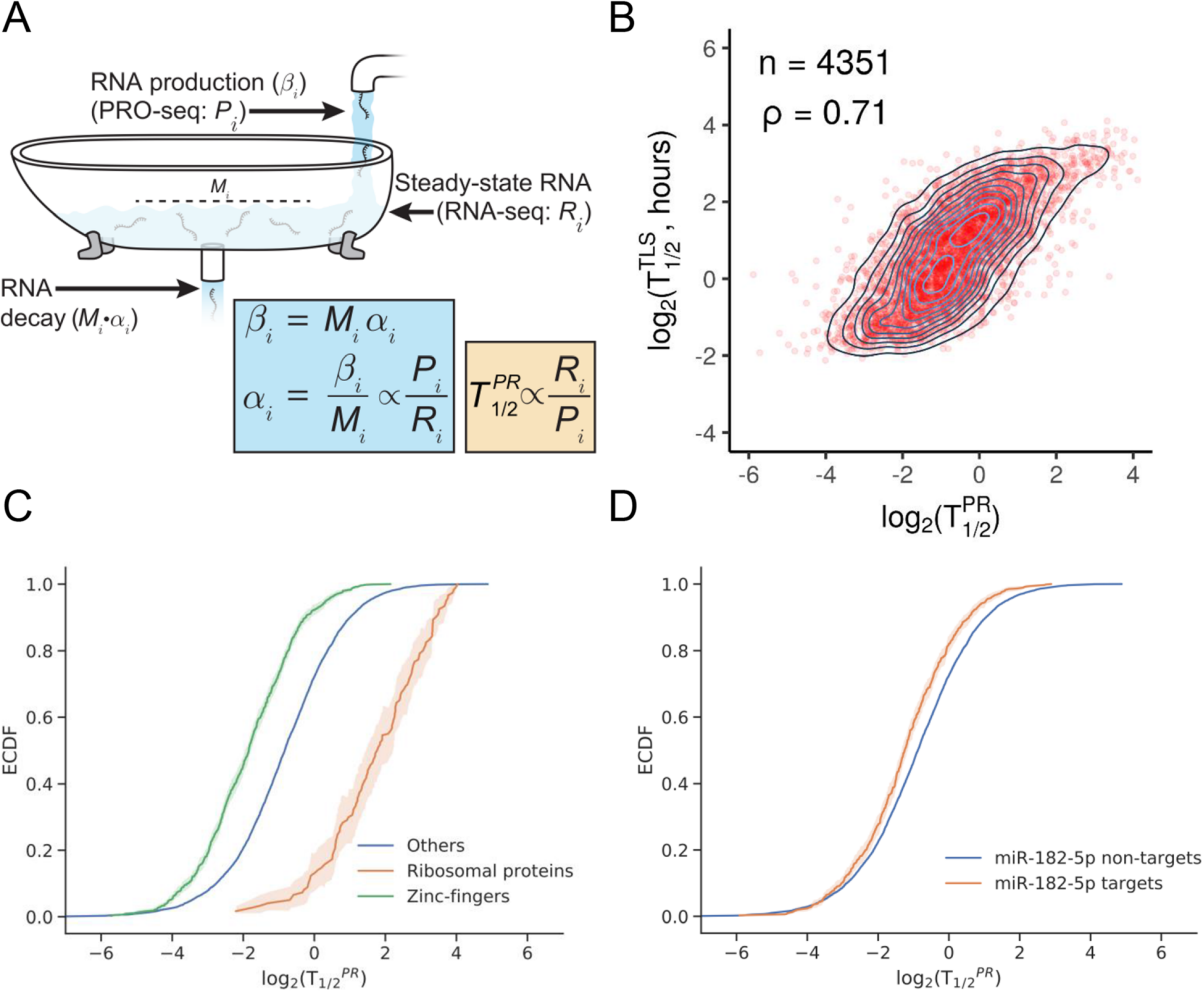
**a** Illustration of dynamic equilibrium between production and decay of RNA. PRO-seq (*P*_*i*_) can be used to measure production and RNA-seq (*R*_*i*_) to measure the resulting equilibrium RNA concentration. At steady state, the production and decay rates must be equal, allowing for estimation of a quantity proportional to RNA half-life (*T*_1/2_^*PR*^) by the ratio *R*_*i*_/*P*_*i*_ (see the “Methods” section). Illustration adapted from [33]. **b** Scatter plot with density contours for (log_2_) half-lives estimated by the PRO-seq/RNA-seq method (*T*_1/2_^*PR*^, *x*-axis) vs. those estimated by TimeLapse-seq (Schofield et al. [20]) (*T*_1/2_^*TLS*^, *y*-axis) for 4351 TUs assayed by both methods in K562 cells. The *T*_1/2_^*PR*^ values are unit-less, whereas the *T*_1/2_^*TLS*^ values are expressed in hours. *ρ* = Spearman’s rank correlation coefficient. **c** Empirical cumulative distribution functions (eCDFs) for (log_2_) estimated RNA half-lives, *T*_1/2_^*PR*^, for ribosomal proteins (*n* = 119), zinc-finger proteins (*n* = 827), and other genes (*n* = 7216; *p* < 3.99e−15, Kolmogorov–Smirnov [K-S] test). **d** Similar eCDFs for mRNAs predicted to be targets (*n* = 668) of miR-182-5p vs. non-targets (*n* = 7494, *p* = 4.75e−8, K-S test). In **c** and **d**, shading indicates 95% confidential intervals as estimated from 1000 bootstrap replicates

As noted above, a quantity proportional to RNA half-life can be approximated in a straightforward manner from measurements of transcription rate and steady-state RNA concentration under equilibrium conditions [21, 22, 32]. Briefly, if *β*_*i*_ is the rate of production of new RNAs for each TU *i*, *α*_*i*_ is the per-RNA-molecule rate of decay, and *M*_*i*_ is the number of RNA molecules, then, at steady state, *β*_*i*_ = *α*_*i*_*M*_*i*_, and the decay rate can be estimated as *α*_*i*_ = *β*_*i*_/*M*_*i*_ (see Fig. 2a and the “Methods” section). If we assume that *β*_*i*_ is approximately proportional to the normalized PRO-seq read counts for *i*, denoted *P*_*i*_, and *M*_*i*_ is proportional to the normalized RNA-seq read counts, denoted *R*_*i*_, then the ratio *P*_*i*_/*R*_*i*_ is an estimator for a quantity proportional to the decay rate, and its inverse, *T*_1/2,*i*_^*PR*^ = *R*_*i*_/*P*_*i*_, is an estimator for a quantity proportional to RNA half-life. As noted, the use of PRO-seq, rather than intronic read counts, for the measure of transcription has a number of advantages, including applicability to intron-less TUs and increased sensitivity for TUs expressed at low levels.

Following this approach, we estimated *T*_1/2_^*PR*^ values for TUs from across the genome using the PRO-seq and RNA-seq data for K562 cells. To validate our estimates, we compared them with estimates of RNA half-life for K562 cells from TimeLapse-seq [20], a recently published method based on chemical conversion of 4sU. We compared our estimates of half-life with those from TimeLapse-seq (denoted *T*_1/2_^*TLS*^) at 4351 genes measured by both methods. We found that the two sets of estimates were reasonably well correlated (Spearman’s *ρ* = 0.71 Fig. 2b), especially considering the substantial differences in experimental protocols and the generally limited concordance of published half-life estimates across experimental methods [6, 15]. By contrast, estimates based on intronic reads showed much poorer agreement with TimeLapse-seq (*ρ* = 0.47; Additional file 2: Figure S9), although it is worth noting that the correction for RNA processing introduced by Alkallas et al. [21] could not be applied in our case, because it requires a comparison of two conditions. We found that our estimated *T*_1/2_^*PR*^ values were significantly shifted toward lower values for zinc finger proteins (Fig. 2c), many of which play key regulatory roles, and toward higher values for ribosomal proteins, which are representative of “housekeeping” genes. We also found that the predicted targets of numerous miRNAs, including the well-studied miR-182 (Fig. 2d) [34], have significantly reduced stability (see Additional file 2: Figure S10 for additional examples).

As further validation, we extended our comparison to include estimates of RNA half-life for K562 cells based on TT-seq [35], SLAM-seq [36], and the method of Mele et al. [37], focusing on 3449 protein-coding genes for which estimates from all methods are available. In general, all methods show significant but somewhat modest levels of correlation in their half-life estimates, ranging from a high value of Spearman’s *ρ* = 0.80 for the TimeLapse-seq and Mele et al. [37] methods to a low of *ρ* = 0.51 for TT-seq and our method (Additional file 2: Figure S11). We attribute these differences in correlation to a variety of both technological and conceptual differences among methods (see the “Discussion” section). Finally, we explicitly adjusted our estimates of relative half-life for elongation rate, and found that the correlation with other methods did not improve (Additional file 2: Figure S12).

### Properties of transcription units that are predictive of RNA stability

**Fig. 3:**
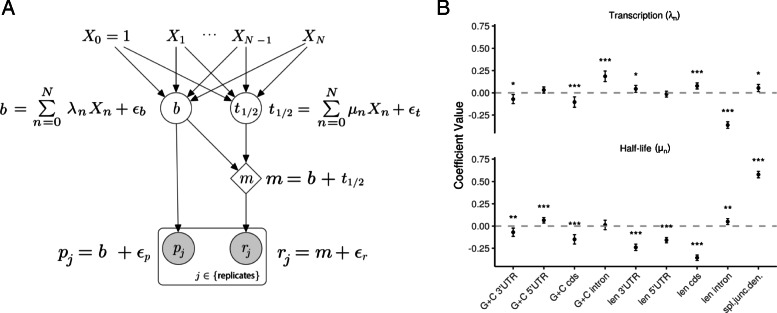
Features of transcription units (TUs) that are predictive of transcription rate and RNA half-life. **a** Structural equation model (SEM) describing the effects of an arbitrary collection of TU features (*X*_1_, …, *X*_*N*_, with intercept term *X*_0_ = 1) on transcription rate (*b*) and half-life (*t*_1/2_), as well as the downstream impact on mRNA concentration (*m*), normalized PRO-seq (*p*), and normalized RNA-seq (*r*) read counts. The model is linear in logarithmic space, with unmodeled variation accounted for as Gaussian noise (*ɛ*_*b*_, *ɛ*_*t*_, *ɛ*_*p*_, and *ɛ*_*r*_; see the “Methods” section). The coefficients for transcription rate (*λ*_*n*_) and half-life (*μ*_*n*_) are estimated by maximum likelihood, assuming independence of replicates and pooling data from all TUs of the same class. **b** Estimated values for coefficients for transcription (*λ*_*n*_; top) and half-life (*μ*_*n*_; bottom) for various features of interest. Results are for intron-containing mRNAs (see Additional file 2: Figures S13 & S14 for other classes). Features considered for each TU: G+C 3′UTR, GC content in 3′UTR; G+C 5′UTR, GC content in 5′UTR; G+C cds, GC content in coding region; G+C intron, GC content in intron(s); len 3′UTR, length of 3′UTR; len 5′UTR, length of 5′UTR; len cds, total length of coding region; len intron, total length of intron(s); spl. junc. dens., number of splice junctions divided by mature RNA length. Error bars represent ± 1.96 standard error, as calculated by the “lavaan” R package [39]. Significance (from *Z*-score): *p < 0.05; **p < 0.005; ***p < 0.0005

To reveal potential determinants of RNA stability, we sought to identify features of TUs that were predictive of our estimated RNA half-lives. We focused on the mRNA and lincRNA classes, for which we could identify the most informative features. Anticipating an effect from splicing [5, 38], we focused our analysis on intron-containing TUs. We considered nine different features related to splicing patterns, transcript length, and G+C content (Fig. 3 and Additional file 2: Figures S13 & S14). In previous studies of this kind, investigators have examined the correlation of each feature with half-life, either individually or together in a multiple regression framework. By construction, however, *T*_1/2_^*PR*^ will tend to be statistically correlated with features predictive of transcription regardless of their true influence on half-life. Therefore, we instead made use of a structural equation model (SEM) [39] that explicitly describes the separate influences of features on transcription and half-life, and the contributions of both to RNA abundance (see the “Methods” section and Fig. 3a).

Our analysis revealed positive correlations with half-life of both splice junction density and total intron length, for intron-containing mRNAs and lincRNAs (Fig. 3b; Additional file 2: Figure S13), although the correlation with splice junction density was not statistically significant in lincRNAs. The observation regarding splice junction density is consistent with previous reports for mRNAs [5, 38, 40, 41] and lincRNAs [42], as well as with the general tendency for intron-containing TUs to be more stable than intron-less TUs (Additional file 2: Figure S15). The correlation with intron length is intriguing but could be an artifact of increased elongation rates in long introns (see below and the “Discussion” section). We also observed several patterns having to do with G+C content and length that are difficult to interpret owing to the complex correlations of these features with CpGs, transcription, splicing, and RNA half-life. Nevertheless, we found that several features had coefficients of opposite sign for transcription and half-life (e.g., 3′UTR, CDS, and intron length), which could be driven, in part, by stabilizing selection on RNA levels (see the “Discussion” section).

To evaluate the degree to which these findings were influenced by elongation rate, we repeated the SEM analysis for a subset of genes (*n* = 1429) also analyzed by Veloso et al. [31], using an updated estimate of transcription rate that explicitly corrected for the estimated elongation rates of these genes (see the “Methods” section). We found that most of the results above held up under this analysis, with the main exception being the positive correlation between intron length and RNA half-life (Additional file 2: Figure S16). This finding could be an artifact of elongation rate in our uncorrected analysis because there is evidence of increased elongation rate (which would be perceived as reduced PRO-seq signal, and hence increased RNA-seq/PRO-seq ratio) in long introns [43]. We also observed some differences in the associations with G+C content.

As further validation, we performed a similar analysis using estimates of half-life based on TT-seq [35], SLAM-seq [36], and the study of Mele et al. [37], focusing on 3418 genes for which features and estimates were available from all methods (Additional file 2: Figure S17). In these cases, we did not have separate measures of transcription and steady-state RNA abundance, so in place of the SEM analysis, we performed multiple linear regression (MLR) using the same features as covariates and the estimated half-lives from each of these other studies as outcomes. For comparison, we repeated the analysis of our half-life estimates also by MLR. In general, we found that the observed trends were similar across all methods. The major exception was 3′UTR length, where the other methods found a positive correlation instead of the negative correlation observed with our method. It is possible that this difference might also reflect a bias in our estimates from elongation rate, which has been observed to decrease near the 3′ ends of genes [8]. However, other studies have also noted a negative correlation between 3′UTR length and half-life, possibly related to the presence of miRNA or RBP binding sites [4, 5, 44].

### DNA sequence correlates of RNA stability

**Fig. 4:**
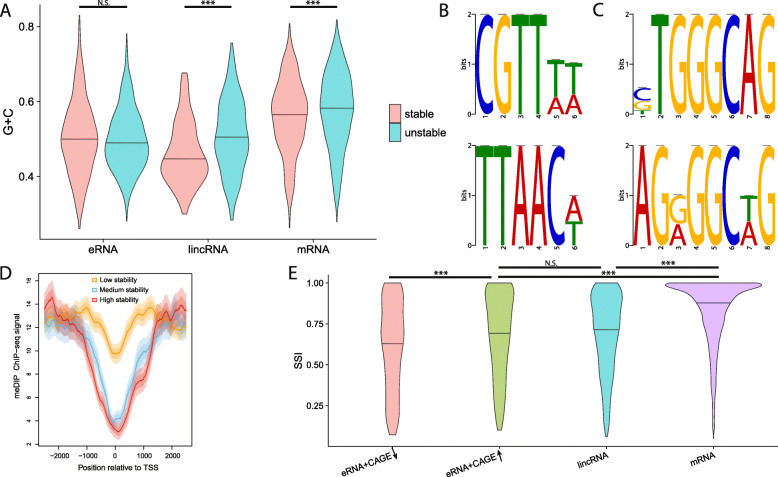
DNA-sequence, methylation, and RNA-binding-protein correlates of RNA stability near the TSS. **a** Distribution of G+C content (*y*-axis) for the 20% most (red) and least (blue) stable TUs, according to our estimated half-life (*T*_1/2_^*PR*^), in enhancer RNAs (eRNA, stable: *n* = 510; unstable: *n* = 510), lincRNAs (stable: *n* = 91; unstable: *n* = 198), and mRNAs (stable: *n* = 919; unstable: *n* = 2146). **b**, **c** Two of the most significantly enriched DNA sequence motifs in stable (**b**) and unstable (**c**) mRNAs. **d** Signal for MeDIP-measured DNA methylation for low-, medium-, and high-stability mRNAs (see the “Methods” section) as a function of distance from the TSS. Solid line represents mean signal and lighter shading represents standard error and 95% confidence interval. **e** Distribution of sequence stability index (SSI) based on U1 and polyadenylation sites (see the “Methods” section) for eRNAs (*n* = 1020), lincRNAs (*n* = 989), and mRNAs (*n* = 10,728). Separate plots are shown for eRNAs with low (*n* = 510) and high (*n* = 510) CAGE support, suggesting low and high stability, respectively. Significance of comparisons in **a** and **e** (from Mann–Whitney *U* test): *p < 0.01; **p < 0.001; ***p < 0.0001; N.S., not significant

Our estimates of RNA half-life for both coding and noncoding TUs provide an opportunity to better characterize DNA sequence correlates of RNA stability near transcription start sites (TSSs) [5, 28, 45, 46]. We tested for associations between half-life and DNA words (*k*-mers) of various lengths near the TSS (see the “Methods” section), but we found that the observed trends were predominantly driven by G+C content, with A+T-rich *k*-mers being enriched, and G+C-rich *k*-mers being depleted, in stable transcripts relative to unstable transcripts (Fig. 4a; Additional file 2: Figures S18–S20). Using the discriminative motif finder DREME [47], we identified several A+T-rich motifs associated with stable transcripts, and several G+C-rich motifs associated with unstable transcripts (Fig. 4b, c). Finally, we expanded our set of TUs to include previously identified eRNAs from K562 cells [28] (see the “Methods” section), and found, interestingly, that stable eRNAs were slightly enriched, rather than depleted, for G+C-rich sequences close to the TSS (Fig. 4a; Additional file 2: Figure S20). This trend was most strongly associated with CpG dinucleotides within 400 bp of the TSS (Additional file 2: Figure S21).

The atypical patterns around CpG dinucleotides raise the possibility of an association with DNA methylation near the TSS. We therefore compared the methylation patterns of TUs exhibiting low, medium, or high levels of RNA stability, summarizing these patterns with meta-plots of average signal of the methylated DNA immunoprecipitation (MeDIP-seq) assay in K562 cells [48, 49] as a function of distance from the TSS (see the “Methods” section). We found that the medium- and high-stability TUs exhibited similar patterns of methylation, but the low-stability TUs show a clear enrichment (Fig. 4d). A similar trend was evident for lincRNAs (Additional file 2: Figure S22). These observations suggest the possibility of epigenomic as well as DNA sequence differences associated with RNA stability, as we explore further below.

### U1 and polyadenylation sites have limited predictive power for stability

We also directly tested for the possibility that differences in RNA half-life could reflect the presence or absence of either U1 binding sites (5′ splice sites) or polyadenylation sites (PAS) downstream of the TSS. Comparisons of (stable) protein-coding TUs and (unstable) upstream antisense RNA (uaRNA) TUs have revealed significant enrichments for proximal PAS in uaRNAs, suggesting that they may lead to early termination that triggers RNA decay. These studies have also found significant enrichments for U1 binding sites in protein-coding TUs, suggesting that splicing may play a role in enhancing RNA stability [45, 46]. In previous work, we showed that these trends generalize to eRNAs as well. In particular, we found that a hidden Markov model (HMM) that distinguished between the occurrence of a PAS prior to a U1 site and the occurrence of a U1 site prior to a PAS could classify TUs as unstable or stable, respectively, with fairly high accuracy [28].

We applied this HMM (see the “Methods” section) to our mRNA and lincRNA TUs and tested whether our DNA sequence-based predictions of stability (as measured by a sequence stability index, or SSI) were predictive of our estimated *T*_1/2_^*PR*^ values. We also computed the SSI for the eRNAs identified from PRO-seq data and classified as stable or unstable based on CAGE data. We found that the mRNAs had the highest SSI, followed by lincRNAs, and then eRNAs (Fig. 4e), as expected. Interestingly, however, the subset of eRNAs that we find to be stable based on CAGE data also show elevated SSIs, roughly on par with lincRNAs. In addition, intron-containing lincRNAs have significantly higher SSIs than intron-less lincRNAs, although there was little difference in intron-containing and intron-less mRNAs (Additional file 2: Figure S23). Moreover, within each of the mRNA and lincRNA groups, we found that the SSI changed relatively little as a function of *T*_1/2_^*PR*^, suggesting that the HMM had almost no predictive power for true RNA stability within these classes (Additional file 2: Figures S24 & S25). These observations suggest that, whereas the U1 and PAS sequence signals do seem to distinguish broad classes of TUs with different levels of stability—namely mRNAs, eRNAs, and uaRNAs—and the same signals are useful in distinguishing stable and unstable eRNAs, other factors likely dominate in determining gradations of stability within the mRNA and lincRNA classes (see the “Discussion” section).

### Additional epigenomic correlates of RNA stability

**Fig. 5:**
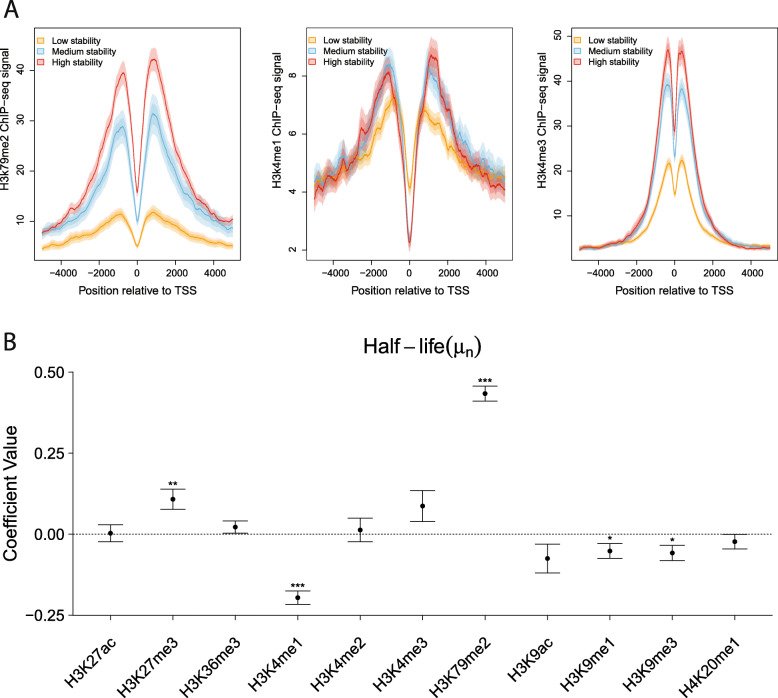
Histone-modification correlates of RNA stability. **a** ChIP-seq signal for H3K79me2 (*left*), H3K4me1 (*middle*), and H3K4me2 (*right*) for low-, medium-, and high-stability mRNAs (see the “Methods” section ) as a function of distance from the TSS. Results are for intron-containing mRNAs matched by normalized PRO-seq signal. Solid line represents mean signal and lighter shading represents standard error and 95% confidence interval. **b** Estimated SEM coefficients for half-life (*μ*_*n*_) for 11 histone modifications, as assayed by ChIP-seq in the 500 bases immediately downstream of the TSS, also for intron-containing mRNAs (see the “Methods” section; see Additional file 2: Figures S28-S30 for additional results). Error bars and significance are as in Fig. 3b

Finally, we asked whether other epigenomic marks such as histone modifications correlate with RNA stability. Histone modifications are primarily associated with transcriptional activity or repression, but there is increasing evidence that events occurring before or during transcription can be associated with post-transcriptional processes and RNA stability [50–54] (see the “Discussion” section). Similar to the methylation analysis above (Fig. 4d), we produced meta-plots showing the average ChIP-seq signal in K562 cells as a function of distance from the TSS for 11 different common histone modifications [48], separately for low-, medium-, and high-stability classes of expression-matched intron-containing mRNAs (see the “Methods” section). While some of these histone modifications did not differ substantially across stability classes, such as H3K9me1 and H3K9me3, several did show clear relationships with estimated RNA half-life (Additional file 2: Figures S26 & S27). For example, H3K79me2, which is associated with transcriptional activity, gives a substantially higher signal in stable transcripts than in unstable ones, particularly in peaks about 1 kb from the TSS (Fig. 5a). A similar pattern is observed for H3K4me2, H3K4me3, H3K9ac, and H3K27ac. The H3K4me1 mark, which is associated with active enhancers, displays a similar pattern far from the TSS but an inverse pattern close to the TSS (Fig. 5a and Additional file 2: Figure S26).

As an alternative strategy for identifying epigenomic correlates of RNA stability while correcting for transcription, we again applied our SEM framework, this time using the 11 histone marks as covariates for estimated RNA half-life and considering the ChIP-seq signals immediately downstream of each TSS (Fig. 5b and Additional file 2: Figure S28). As expected, the strongest correlations were detected with transcription rate, and these generally had the expected sign, for example, with positive correlations for the activation marks H3K27ac, H3K4me1, and H3K4me3, and negative correlations for the repressive marks H3K9me3 and H3K27me3. These patterns were generally consistent between lincRNAs and mRNAs (Additional file 2: Figures S28 & S29), and they did not change substantially as a function of distance from the TSS (Additional file 2: Figures S30 & S31). However, we did additionally identify several significant correlates of half-life. For mRNAs, these were generally consistent with the ones identified from the ChIP-seq meta-plots, for example, with H3K79me2 showing a positive correlation with RNA half-life, and H3K4me1 showing a negative correlation close to the TSS. In general, the estimated coefficients were similar for mRNAs and lincRNAs, but there were some notable differences: for example, the activity mark H3K36me3 shows a negative correlation with RNA half-life in lincRNAs but a weaker and position-dependent positive correlation with mRNA half-life, and the silencing mark H3K9me3 shows a position-dependent positive correlation for lincRNA half-life but a negative correlation for mRNA half-life (Additional file 2: Figures S28 & S29). These divergent patterns could possibly reflect differences in the degree to which splicing is co-transcriptional in mRNAs and lincRNAs [55].

## Discussion

In this article, we have introduced a simple method for estimating the RNA half-lives of TUs from across the genome based on matched RNA-seq and PRO-seq data sets. Like previous methods based on intronic reads, our method assumes equilibrium conditions and produces a relative measure only of half-life. Unlike these methods, however, the use of PRO-seq allows us to interrogate intron-less TUs and TUs that are expressed at low levels. Moreover, even for intron-containing and abundantly expressed genes, the PRO-seq-based measurements appear to be considerably more accurate than those based on intronic reads. Our approach also has a number of advantages in comparison to existing methods for estimating RNA half-lives based on transcriptional inhibition or metabolic labeling. For example, it does not require collecting data in a time course, which enables efficient use of both time and sample material; it can make use of RNA-seq or PRO-seq data generated for other purposes; it is relatively nondisruptive of the biological processes under study; and it can be extended to tissue samples using ChRO-seq [26] (see Additional file 1: Table S1). We have shown that our measurements of relative half-life are useful in a wide variety of downstream analyses.

Our original design for this study was to generate our own PRO-seq and RNA-seq data from the same source of K562 cells, to ensure the data sets were as closely matched as possible. In addition, we produced total rRNA-depleted RNA-seq libraries, rather than poly-A+ libraries, with the goal of improving our sensitivity for noncoding RNAs. For validation, we compared our results with ones based on previously published PRO-seq data [28] and poly-A+ RNA-seq data from the ENCODE project [48]. As it happened, however, we found that the half-life estimates based on these previously published data sets were less variable, showed better agreement with published estimates, and did not differ substantially in sensitivity from the ones based on our newly collected data. Therefore, we have focused on these estimates with our main analyses. In future work, we hope to more systematically compare the attributes of total RNA and poly-A+ libraries [56, 57]. It may also be informative to compare measurements based on RNA extracted from particular cellular compartments, such as the nucleus or the cytoplasm. In general, it may be possible to begin to disentangle the contributions of distinct RNA decay pathways (e.g., 3′→5′ decay, decapping and 5′→3′ decay, nonsense-mediated decay), and their differential effects on distinct classes of RNAs, through such comparisons [58]. In addition, it may be worthwhile to examine how RNA stability varies across conditions or cell types, as most studies so far have only measured RNA stability for a particular cell type under a particular set of conditions.

In a comparison of half-life estimates from several methods that have all been applied to K562 cells, including TimeLapse-seq [20], TT-seq [35], SLAM-seq [36], and the method of Mele et al. [37], we found reasonable agreement across methods, but also some differences (Additional file 2: Figure S11). The average pairwise Spearman's correlation coefficient between sets of estimates was relatively modest at *ρ* = 0.64. It is difficult at this stage to disentangle the sources of the differences among methods. Most likely, they result both from experimental noise and from a combination of more fundamental differences, including whether the estimates are based on steady-state assumptions or time-course measurements, whether transcriptional inhibition or activation is used, how the rate of transcription is assayed, and whether RNA abundance is based on total RNA or polyA+ RNA. These differences may make some methods better for certain classes of TUs than others (e.g., coding vs. noncoding RNAs, lowly vs. highly expressed TUs, intron-containing vs. intron-less TUs, or RNAs that are or are not at equilibrium). More work will be required to clarify the relative strengths and weaknesses of the available methods.

Notably, our method has limited sensitivity for highly unstable transcripts. When half-lives are low, the RNA-seq signal tends toward zero, leading to limited ability to identify gradations of stability. For this reason, we have focused our half-life analysis on genes with fairly strong signals from both assays (PRO-seq > 10 TPM and RNA-seq > 1 TPM; see the “Methods” section). At the same time, similar limitations occur with essentially all of the available assays for half-life estimation, and our approach at least has the advantage that PRO-seq is highly sensitive as a measure of transcriptional activity.

Perhaps a more important limitation of our method is that, strictly speaking, PRO-seq is a measure not of the rate of transcription but of the occupancy of engaged RNA polymerases, which reflects both the rate of transcription and the rate of elongation. The PRO-seq signal along a gene body is analogous to the headlight brightness on a highway at night; an increase in signal can reflect either an increased number of cars entering the highway (analogous to an increased rate of transcription), or a back-up in traffic (analogous to a decreased elongation rate). Consequently, variation in *T*_1/2_^*PR*^ across TUs could in part be driven by variation in elongation rate. We attempted to control for this possibility in several ways. First, we explicitly corrected our estimates of transcription and half-life with previously published estimates of elongation rate for the same cell type [31] (see the “Methods” section). We found that the correction did not improve the correlation of PRO-seq and RNA-seq measurements (Additional file 2: Figure S8), nor did it improve the agreement with independent estimates of half-life (Additional file 2: Figure S12). Second, we repeated our analysis of features predictive of half-life with the corrected estimates and found that it did not substantially alter our results, with one notable exception (Additional file 2: Figure S16; discussed below). Third, we observed that the variation in elongation rate across genes is only about one fifth of the variation in estimated half-lives, indicating that it can account for, at most, a fairly small fraction of the observed variation (Additional file 3: Supplemental Text). We conclude from these analyses that elongation rate does undoubtedly have some impact on our half-life estimates, but overall, the effects appear to be limited. However, more work will be needed to obtain more accurate and more comprehensive estimates of elongation rates, and to fully understand their impact on half-life estimates.

To identify features that are predictive of RNA half-life, we devised a structural equation model (SEM) that explicitly describes the separate effects of each feature on transcription and half-life, as well as the resulting impact on RNA concentrations, PRO-seq, and RNA-seq data. While multivariate regression has been used to identify features associated with RNA stability [5], our analysis is the first, to our knowledge, to attempt to disentangle the separate influences of such features on transcription and RNA stability. It is worth noting that this framework could also be useful for estimators based on intronic reads. The results of the SEM analysis were consistent with previous findings in many respects, particularly regarding the association between RNA splicing and RNA stability. The mechanism underlying this relationship remains unclear, but it is known that the exon junction complex (EJC) remains bound to the mature mRNA after its transport to the cytoplasm and it has been proposed that EJC components may protect the mRNA from decay [5, 41]. In addition to the previously reported positive correlation of splice junction density and RNA half-life, we also observed a positive correlation between intron length and half-life. This observation could potentially indicate that RNA stability is enhanced by recursive splice sites [59] or extended contact with the spliceosome in long introns. However, we could not confirm this finding after our correction for elongation rate using a subset of our full gene set, and it may therefore be an artifact of increased elongation rates in long introns. More work will be needed to confirm or reject this association.

It has recently been reported that U1 binding sites are enriched, and polyadenylation sites are depleted, downstream of the TSS in stable mRNAs relative to unstable upstream antisense RNAs (uaRNAs) and enhancer RNAs (eRNAs), suggesting that RNA stability is determined, in part, by the DNA sequence near the TSS. In this study, we tested not only whether this “U1-PAS axis” could distinguish TUs in stable classes (mRNAs) from those in unstable classes (uaRNAs and eRNAs) but also how predictive it is of half-life within these classes. We confirmed that a U1-PAS-based “sequence stability index” (SSI) is generally elevated for mRNAs, intermediate for lincRNAs, and reduced for eRNAs. Furthermore, this SSI can distinguish between more and less stable eRNAs, as quantified using CAGE (Fig. 4e). Somewhat surprisingly, however, we found that the SSI has essentially no predictive power for relative RNA stability within the generally more stable mRNA and lincRNA classes (Additional file 2: Figures S24 & S25). One possible explanation for this observation is that the U1-PAS axis determines a kind of early “checkpoint” for stable transcripts—for example, by ensuring that premature transcriptional termination is avoided—but that once a transcript has cleared this checkpoint, these DNA sequence features are no longer relevant in determining RNA stability. Instead, the relative stability of mRNAs and lincRNAs may be predominantly determined by splicing-related processes, binding by miRNAs or RBPs, or other post-transcriptional phenomena. More work will be needed to fully understand the mechanistic basis of these differences in stability.

Some of the associations that we observed with half-life concerned G+C content, but these observations are generally difficult to interpret. Indeed, even the comparatively straightforward question of the relationship between G+C content and transcriptional activity has a long and contradictory literature, with several studies finding correlations between them [60–62], but others claiming that the relationship between G+C and transcription is weak, at best, once confounding factors such as genomic context are properly accounted for [63, 64]. Sharova et al. [5] identified a fairly pronounced negative correlation between RNA stability and the prevalence of CpGs in the 5′UTR, which is not supported by our analysis—although we interrogated only G+C content, not CpGs, in the 5′UTR. These authors raised the intriguing hypothesis this correlation may reflect the activity of splicing-associated methyl CpG-binding proteins [65], but to our knowledge, this idea has not been tested experimentally. In any case, it seems unlikely that the complex relationships among G+C content, CpGs, transcription, RNA stability, and downstream effects such as translational efficiency can be fully disentangled through post hoc statistical analyses. Instead, this effort will require experiments that directly perturb individual features of interest and separately measure the effects on a variety of processes.

There is now substantial evidence for connections between events that occur before or during transcription and a variety of post-transcriptional processes, some of which impact RNA stability. In addition to the apparent enhancement of RNA stability by splicing, there is now evidence that some RNA-binding proteins having roles in RNA export and stability are recruited to the RNA in a promoter-dependent manner [66–69]. Similarly, co-transcriptional processes such as polyadenylation and capping appear are linked to RNA stability [51]. It was also recently shown that disrupting transcription rates could lead to enhanced m6A deposition, shortened polyA tails, and reduced RNA stability [52, 53]. With these observations in mind, we looked for epigenomic correlates of stability. We identified several histone modifications that are significantly associated with increased or decreased half-life, but we cannot rule out the possibility that these correlations reflect indirect relationships with confounding variables not considered here. However, the effect is quite strong for certain marks (such as H3K79me2 and H3K4me2) and it is apparent both in direct comparisons of PRO-seq-matched TUs (Fig. 5a) and in the SEM setting (Fig. 5b). It therefore seems plausible that it has a direct mechanistic basis, perhaps involving factors that interact both with DNA-bound nucleosomes and the spliceosome. Some divergent patterns for mRNAs and lincRNAs (Additional file 2: Figure S28) suggest the possibility of differences in these splicing-associated processes. Additional work will be needed to test these hypotheses.

One general pattern that emerges from the SEM analysis of histone modifications is that the coefficients for transcription and half-life are often different from zero in opposite directions (Additional file 2: Figures S28-S31). This trend of anti-correlation was less prominent with the TU features, but we did observe it with CDS, intron, and 3′UTR length (Fig. 3b). A possible explanation for this pattern is that it is, at least in part, a reflection of stabilizing selection on gene expression. If selection tends to favor a particular RNA level for each TU, then mutations that increase transcription may tend to be compensated for by mutations that decrease RNA stability, and vice versa. Thus, stabilizing selection might result in a tendency for features that are positively correlated with one measure (transcription or stability) to be negatively correlated with the other. Notably, this type of hypothetical causal interrelationship between transcription and stability is not considered in our SEM, nor in any other statistical model of which we are aware. As a result, it may be difficult to distinguish correlations that have a direct, mechanistic basis (say, relating to transcription) from their indirect “echoes” (say, relating to half-life) resulting from evolutionary constraint. Despite this potential limitation, our framework remains useful for identifying potentially interesting correlations, whose mechanistic underpinnings can then be further investigated through direct experimental perturbation.

## Conclusions

We introduce a novel approach for estimating the relative half-lives of individual RNAs using PRO-seq and RNA-seq. We develop a structural equation model and test multiple features for their associations with RNA stability after controlling for the effects on transcription. Together, our estimation method and systematic analysis shed light on the pervasive impacts of RNA stability on cellular RNA concentrations.

## Methods

### PRO-seq and RNA-seq data preparation and processing

Our main analysis is based on PRO-seq data for K562 [28] and HeLa [70] cell lines as well as RNA-seq data from the ENCODE project [48, 71] (ENCSR000AEM for K562, ENCSR000CPR for HeLa). For comparison, we also sequenced new PRO-seq (*n* = 2) and RNA-seq (*n* = 4) libraries, generated from cells grown in the same flask under the same conditions. Human K562 cells were cultured using standard cell culture procedures and sterile techniques. The cells were cultured in RPMI-1640 media supplemented with 10% fetal bovine serum (FBS) and 1% penicillin/streptomycin. For PRO-seq, 3′ and 5′ adapters were ligated as described [26] followed by library preparation as previously published [72]. Sequencing was done by Novogene on a HiSeq instrument with paired-end reads of 2 × 150 bp. For RNA-seq, total RNA was extracted using the Trizol method (see https://assets.thermofisher.com/TFS-Assets/LSG/manuals/trizol_reagent.pdf), followed by rRNA depletion using the Ribozero HMR Gold kit. Libraries were prepared using the NEB kit with TruSeq RNAseq adaptors. Single-end sequencing (length = 75) was performed on a NextSeq500 instrument by the RNA Sequencing Core at the College of Veterinary Medicine, Cornell University. Sequencing data is deposited on GEO under accession GSE153200.

### Read mapping and transcript abundance estimation

Raw data files in fastq format were trimmed using Cutadapt [73] with parameters -j 0 -e 0.10 --minimum-length=10. Reads were then aligned using HISAT2 [74] with default parameters (hisat2 --threads 4 -x {index} -U {input.reads} -S {output} --summary-file {log}). We used the GRCh38/hg38 reference genome and the associated GENCODE gene annotations. HTSeq [75] was used for read counting for RNA-seq and PRO-seq. For the purposes of read counting with PRO-seq, we omitted the first 500 bases downstream of the TSS and 500 bases upstream of TES to avoid a bias in read counts from promoter-proximal pausing and polymerase deceleration. Finally, we normalized read counts by converting them to transcripts per million (TPM) [76] based on the length of each TU.

### Estimation of RNA half-life from RNA-seq and PRO-seq data

We assume a constant rate of production of new RNAs, *β*_*i*_; a constant per-RNA-molecular rate of decay, *α*_*i*_; and a number of RNA molecules, *M*_*i*_. At steady state, *β*_*i*_ = *α*_*i*_*M*_*i*_; therefore, the decay rate can be estimated as *α*_*i*_ = *β*_*i*_/*M*_*i*_, and the half-life as *T*_1/2_ = ln(2)/*α*_*i*_ = ln(2) × *M*_*i*_/*β*_*i*_. We further assume that the normalized PRO-seq read counts (omitting regions near the TSS and TES) are proportional to the rate of production of new RNAs, *P*_*i*_ ∝ *β*_*i*_, and that the normalized RNA-seq read counts are proportional to the number of RNA molecules, *R*_*i*_ ∝ *M*_*i*_. Therefore, *T*_1/2_ ∝ *R*_*i*_/*P*_*i*_. We define our unit-less estimator of half-life as *T*_1/2_^*PR*^ = *R*_*i*_/*P*_*i*_, where *PR* denotes a PRO-seq/RNA-seq-based estimator. Notice that these unit-less *T*_1/2_^*PR*^ values can be compared across experiments only up to a proportionality constant, unless the raw read counts have been appropriately normalized. To compare our PRO-seq-based approach with an approach based on intronic reads, we repeated the estimation using normalized intron reads instead of PRO-seq read counts to represent the transcription rate.

### Correction for elongation rate in PRO-seq vs. RNA-seq correlation and half-life estimations

A potential confounding factor in the comparison of normalized read counts for PRO-seq and RNA-seq is elongation rate. Because PRO-seq read depth reflects a combination of transcription initiation rates and elongation rates [30, 77], some reduction in correlation with RNA-seq could reflect variability across TUs in elongation rate. We investigated this possibility by using published measurements of elongation rate for the same K562 cell type [31], focusing on ~2000 genes that overlap our set. We explicitly adjusted for the estimated elongation rates by multiplying them by the PRO-seq abundance across gene bodies, under the assumption that the PRO-seq density is proportional to the transcription rate divided by the elongation rate. The corrected PRO-seq abundance was then used for comparison with RNA-seq, for half-life estimation, and for the SEM analysis.

### Structural equation model

To separate the effects of TU features on decay from the effects on transcription, we developed a SEM using the “lavaan” R package [39]. Let *X*_*n*_ be the *n*th feature associated with a TU. We assume that the logarithms of this TU’s transcription rate and half-life, i.e., *b* = log *β* and *t*_1/2_ = log *T*_1/2_^*PR*^, are linear combinations of the *X*_*n*_’s and a TU-level random effect: *b* $={\sum}_{n=0}^N$ *λ*_*n*_*X*_*n*_ + *ε*_*b*_ and *t*_1/2_ $={\sum}_{n=0}^N$ *μ*_*n*_*X*_*n*_ + *ε*_*t*_ where *ϵ*_*b*_ ∼ *N*(0, *σ*_*b*_) and *ϵ*_*t*_ ∼ *N*(0, *σ*_*t*_) are independent Gaussian random variables explaining all variation not attributable to known features. Assuming a fixed value *X*_0_ = 1 for all genes, the parameters *λ*_0_ and *μ*_0_ can be interpreted as intercepts whereas *λ*_*n*≠0_ and *μ*_*n*≠0_ are regression coefficients indicating the contributions of feature *n* to transcription rate and half-life, respectively.

According to the model derived above, at steady state, *T*_1/2_^*PR*^∝ *M/β*, where *M* is the number of RNA molecules; therefore, *m* = log *M* is given by *m* = *b* + *t*_1/2_ + *C*, where *C* is an arbitrary constant that can be ignored here because it does not affect the estimation of regression coefficients. Denoting *p*_*j*_ = log *P*_*j*_ and *r*_*j*_ = log *R*_*j*_ as the logarithms of the PRO-seq and RNA-seq measurements in replicate *j*, respectively, we assume *p*_*j*_ ~ *b* + *ε*_*p*_ and *r*_*j*_ ~ *m* + *ε*_*r*_ where *ϵ*_*p*_ ∼ *N*(0, *σ*_*p*_) and *ϵ*_*r*_ ∼ *N*(0, *σ*_*r*_) are independent Gaussian random variables describing the noise in PRO-seq and RNA-seq experiments, respectively. Finally, we assume that all observations are independent across TUs. With these assumptions, and pooling information across TUs of the same class, we can estimate separate regression coefficients for transcription rates (*λ*_*n*_) and half-life (*μ*_*n*_) for all features by maximum likelihood.

### Transcription unit features

Transcription unit (TU) sequences were downloaded from BioMart using the R package biomaRt [78, 79]. We considered only one isoform per annotated gene, i.e., the most abundant transcript determined by Salmon [80]. Features based on properties of DNA sequences (e.g., G+C content) were then extracted using Biopython [81]. The intron length was set equal to the transcript length minus the total exon length. The splice junction density was set equal to the intron number divided by the mature RNA length.

### eRNA analysis

We used eRNAs identified from our previous GRO-cap analysis in K562 cells [28] restricting our analysis to putative eRNAs with divergent transcription [27] that fell at least 1 kb away from annotated genes (*n* = 21,816). To measure steady-state RNA levels, we used CAGE in place of RNA-seq owing to its greater sensitivity. We used the Nucleus PolyA and Non-polyA CAGE libraries from ENCODE (GEO accession number GSE34448). To measure transcription rates, we used PRO-seq data from same study [28]. For the stability analysis, we eliminated TUs having no mapped CAGE reads, and then selected the top 10% by CAGE/PRO-seq ratio as “stable” and the bottom 10% as “unstable.” These stable and unstable groups were then matched by PRO-seq signal (*n* = 510).

### DNA word enrichments

We considered all DNA words (all possible combinations of A, C, G, T) of sizes *k* ∈ {2, 3, 4}. For each word *w*, we counted the total number of appearances in our set of stable TUs (top 20% by *T*_1/2_^*PR*^), denoted *c*_*s,w*_, and the total number of appearances in unstable TUs (bottom 20% by *T*_1/2_^*PR*^), denoted *c*_*u,w*_. These counts were collected in 1 kb windows beginning at various distances downstream of the TSS (0, 500, 1000, and 1500 bp). The enrichment score for each word *w* and each window position was then computed as log_2_(*c*_*s,w*_/*c*_*u,w*_). A positive value of this score indicates an enrichment, and a negative score indicates a depletion in stable TUs relative to unstable TUs. For eRNAs, we used a similar procedure but with 400 bp windows at distances of 0, 200, 400, and 600 bp from the TSS.

### Motif discovery

For motif discovery, we used the discriminative motif finder “DREME” [47] with default parameters (core width ranging from 3 to 7). For the stable motifs, we used the top 20% of TUs by *T*_1/2_^*PR*^ as the primary sequences and the bottom 20% as the control sequences. For the unstable motifs, we reversed the primary and control sequences.

### Sequence stability index

We define the SSI to be the probability that a TU is “stable” based on our previously published U1-PAS hidden Markov model (HMM) [28]. Briefly, the HMM identifies a TU sequence as “stable” if either (1) it has a U1 splicing motif upstream of a PAS motif or (2) it lacks both a PAS motif and a U1 splicing motif, as detailed by Core et al. [28]. We applied the HMM to the first 1 kb of sequence downstream of the annotated TSS and calculated the SSI as 1 minus the probability the TU is unstable, as output by the program. An implementation of the HMM is available at https://github.com/Danko-Lab/stabilityHMM.

### Matching by PRO-seq expression

We used the R package “MatchIt” [82, 83] to match groups of TUs by their normalized PRO-seq read counts (method = “nearset”). In cases of multiple groups, one group was selected as the reference and every other group was matched to that reference group.

### MicroRNA targets analysis

We obtained microRNA targets from TargetScanHuman [84], Release 7.2 (http://www.targetscan.org/vert_72/vert_72_data_download/Predicted_Targets_Info.default_predictions.txt.zip). We used all default predictions of conserved targets for each conserved miRNA family in the database.

### Gene categories

We obtained lists of genes encoding ribosomal proteins and zinc fingers from the HUGO Gene Nomenclature Committee (https://www.genenames.org/).

### Epigenomic analysis

Histone modifications (ChIP-seq; http://hgdownload.soe.ucsc.edu/goldenPath/hg19/encodeDCC/wgEncodeSydhHistone/) and DNA methylation IP (MeDIP; GEO accession number GSM1368906) data were downloaded from the ENCODE consortium [48] as bigwig files annotated to the GRCh37/hg19 reference genome. We partitioned our mRNAs, considering intron-containing TUs only, into five equally sized stability classes based on the estimated *T*_1/2_^*PR*^ values, and then subsampled from classes 1 (low stability), 3 (medium stability), and 5 (high stability) to obtain distributions matched by PRO-seq signal. We then produced meta-plots for each of these three classes showing the average signal of the histone modifications (ChIP-seq) and methylated DNA immunoprecipitation (MeDIP-seq) assays in K562 cells [48, 49] as a function of distance from the TSS. Meta-plots showing the average values of signals of interest across loci (e.g., Figs. 4d and 5a) were produced using the “plotMeta” function from the “Genomation” [85] R package. The input signal was provided in bigwig format, and the loci were defined in bed format. In all cases, the average signal is plotted as a colored line, with uncertainty indicated by the standard error of the mean (darker shading) and 95% confidence intervals (lighter shading) as specified by the “se” parameter.

## Supplementary Information

**Additional file 1: Table S1.** Summary of Advantages of Method.

**Additional file 2: Figure S1.** Correlation of PRO-seq and RNA-seq replicates in K562 cells. **Figure S2.** Correlation of exonic reads from RNA-seq with either PRO-seq or intronic reads. **Figures S3-S4.** Scatter plots of PRO-seq vs. RNA-seq for intron-containing (Figure S3) or intron-less (Figure S4) transcription units in K562 cells. **Figure S5.** Scatter plots of PRO-seq vs. RNA-seq for lincRNAs and protein-coding mRNAs in K562 cells after matching by PRO-seq signal. **Figures S6-S7.** Scatter plots of PRO-seq vs. RNA-seq for transcription units in K562 cells (Figure S6) or HeLa cells (Figure S7). **Figure S8.** Scatter plots of PRO-seq vs. RNA-seq for mRNAs and lincRNAs in K562 cells before and after correcting for elongation rate. **Figure S9.** Intronic half-life vs. TimeLapse-seq half-life. **Figure S10.** Empirical cumulative distribution functions (eCDFs) for estimated half-lives of predicted targets of miR-125-5p and miR-19-3p vs. non-targets. **Figure S11.** Correlation of estimated RNA half-lives under various methods. **Figure S12.** Correlation of PRO-seq-based half-lives (*T*_1/2_^*PR*^, *x*-axis) vs. estimates from TimeLapse-seq (*T*_1/2_^*TLS*^, *y*-axis) after correcting for elongation rate. **Figures S13-S14.** SEM results for features of intron-containing (Figure S13) or intron-less (Figure S14) transcription units in K562. **Figure S15.** Estimated half-lives for intron-containing and intron-less transcription units. **Figure S16.** SEM results for features of intron-containing transcripts in K562 cells, with and without a correction for elongation rate. **Figure S17.** Multiple linear regression (MLR) for features of transcription units versus RNA stability in K562 cells. **Figures S18-S19.** DNA word enrichments in stable transcripts for protein-coding mRNAs (Figure S18) or lincRNAs (Figure S19). **Figure S20.** G+C content in intervals downstream of the TSS for various classes of transcription units. **Figure S21.** DNA word enrichments in stable transcripts for eRNAs. **Figure S22.** DNA methylation in lincRNAs of various stability levels. **Figure S23.** Sequence Stability Index of intron-containing versus intron-less genes. **Figures S24-S25.** Sequence Stability Index (SSI) for mRNAs (Figure S24) or lincRNAs (Figure S25) of various stability classes. **Figures S26-S27.** Histone modification signals for protein-coding mRNAs of various stability classes. Half-live estimations are based on published data (Figure S26) or newly collected data for this study (Figure S27). **Figures S28-S29.** Estimated SEM coefficients for transcription (*λ*_*n*_; top) and half-life (*μ*_*n*_; bottom) for 11 histone modifications. It’s assayed by ChIP-seq in the 500 bases (Figure S28) or 1000-1500 bases (Figure S29) downstream of the TSS. **Figures S30-S31.** Estimated SEM coefficients for transcription (*λ*_*n*_; top) and half-life (*μ*_*n*_; bottom) for 11 histone modifications. It’s based either on published data (Figure S30) or on the newly collected PRO-seq and RNA-seq data (Figure S31).

**Additional file 3.** Variation in Elongation Rate is Insufficient to Explain Variation in Half-Life.

## Data Availability

All data generated or analyzed during this study are included in this published article, its supplementary information files and publicly available repositories. PRO-seq and RNA-seq data generated for this study are deposited on GEO under accession GSE153200. Published PRO-seq data for K562 [28] and HeLa [70] cell lines are retrieved from GEO under accession GSE60456 and GSE100742. Published RNA-seq data are retrieved from the ENCODE project [48, 71] (ENCSR000AEM for K562, ENCSR000CPR for HeLa). The source code used for data analysis and visualization is publicly available via Code Ocean at https://codeocean.com/capsule/7351682/tree/v1.
