# Supplementary material for: Characterizing RNA stability genome-wide through combined analysis of PRO-seq and RNA-seq data
Source: BMC Biol. 2021 Feb 15;19:30. doi: 10.1186/s12915-021-00949-x (PMC7885420; doi:10.1186/s12915-021-00949-x)
Supplement: Supplementary file 1 — Additional file 1: Table S1. Summary of Advantages of Method. [file 12915_2021_949_MOESM1_ESM.pdf]

Supplemental Table 1: Summary of Advantages of Method

Advantage	***Description***
Efficiency of time and material	No need to collect data for full time course, reducing the number of experiments and the amounts of material needed
Reuse of existing data	RNA-seq and PRO-seq data sets can be decoupled. As a result, existing RNA-seq and/or PRO-seq data for adequately matched cells can be reused.
Other applications of data	Newly collected RNA-seq or PRO-seq data can be used for other purposes, e.g., analysis of proximal promoter pausing or identification of active enhancers
Extension to tissue samples	Can be applied to tissue samples using ChRO-seq
High sensitivity	Exploits high sensitivity of PRO-seq to noncoding and other low-abundance transcripts
Nondisruptive	Less disruptive to the biological processes under study than most drugs used for transcriptional inhibition and metabolic labeling. PRO-seq captures the positions of engaged RNA polymerases under the cellular conditions that exist when the experiment commences
Continual improvement	PRO-seq protocol is continually being improved. Current improvements allow PRO-seq libraries to be prepared in one day with comparable difficulty to enriching RNA-seq libraries for 4sU (Kim et al. 2020)
