# Supplementary material for: Characterizing RNA stability genome-wide through combined analysis of PRO-seq and RNA-seq data
Source: BMC Biol. 2021 Feb 15;19:30. doi: 10.1186/s12915-021-00949-x (PMC7885420; doi:10.1186/s12915-021-00949-x)
Supplement: Supplementary file 2 — Additional file 2: Figure S1. Correlation of PRO-seq and RNA-seq replicates in K562 cells. Figure S2. Correlation of exonic reads from RNA-seq with either PRO-seq or intronic reads. Figures S3-S4. Scatter plots of PRO-seq vs. RNA-seq for intron-containing (Figure S3) or intron-less (Figure S4) transcription units in K562 cells. Figure S5. Scatter plots of PRO-seq vs. RNA-seq for lincRNAs and protein-coding mRNAs in K562 cells after matching by PRO-seq signal. Figures S6-S7. Scatter plots of PRO-seq vs. RNA-seq for transcription units in K562 cells (Figure S6) or HeLa cells (Figure S7). Figure S8. Scatter plots of PRO-seq vs. RNA-seq for mRNAs and lincRNAs in K562 cells before and after correcting for elongation rate. Figure S9. Intronic half-life vs. TimeLapse-seq half-life. Figure S10. Empirical cumulative distribution functions (eCDFs) for estimated half-lives of predicted targets of miR-125-5p and miR-19-3p vs. non-targets. Figure S11. Correlation of estimated RNA half-lives under various methods. Figure S12. Correlation of PRO-seq-based half-lives (T1/2PR, x-axis) vs. estimates from TimeLapse-seq (T1/2TLS, y-axis) after correcting for elongation rate. Figures S13-S14. SEM results for features of intron-containing (Figure S13) or intron-less (Figure S14) transcription units in K562. Figure S15. Estimated half-lives for intron-containing and intron-less transcription units. Figure S16. SEM results for features of intron-containing transcripts in K562 cells, with and without a correction for elongation rate. Figure S17. Multiple linear regression (MLR) for features of transcription units versus RNA stability in K562 cells. Figures S18-S19. DNA word enrichments in stable transcripts for protein-coding mRNAs (Figure S18) or lincRNAs (Figure S19). Figure S20. G+C content in intervals downstream of the TSS for various classes of transcription units. Figure S21. DNA word enrichments in stable transcripts for eRNAs. Figure S22. DNA methylation in lincRNAs of various stab [file 12915_2021_949_MOESM2_ESM.pdf]

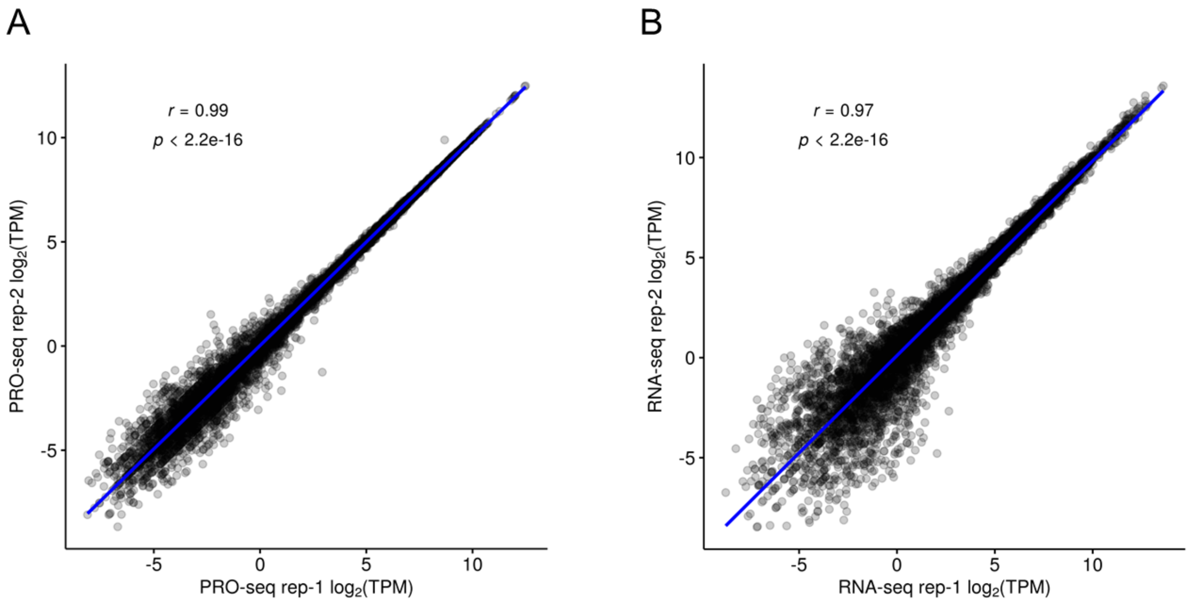

Supplemental Figure 1. Correlation of PRO-seq and RNA-seq replicates in K562 cells. (A) Pearson's correlation coefficient *r* for two replicates assayed by PRO-seq. (B) Pearson's correlations coefficient *r* for two replicates assayed by RNA-seq. In both cases, genes without reads in one or more replicates were excluded.

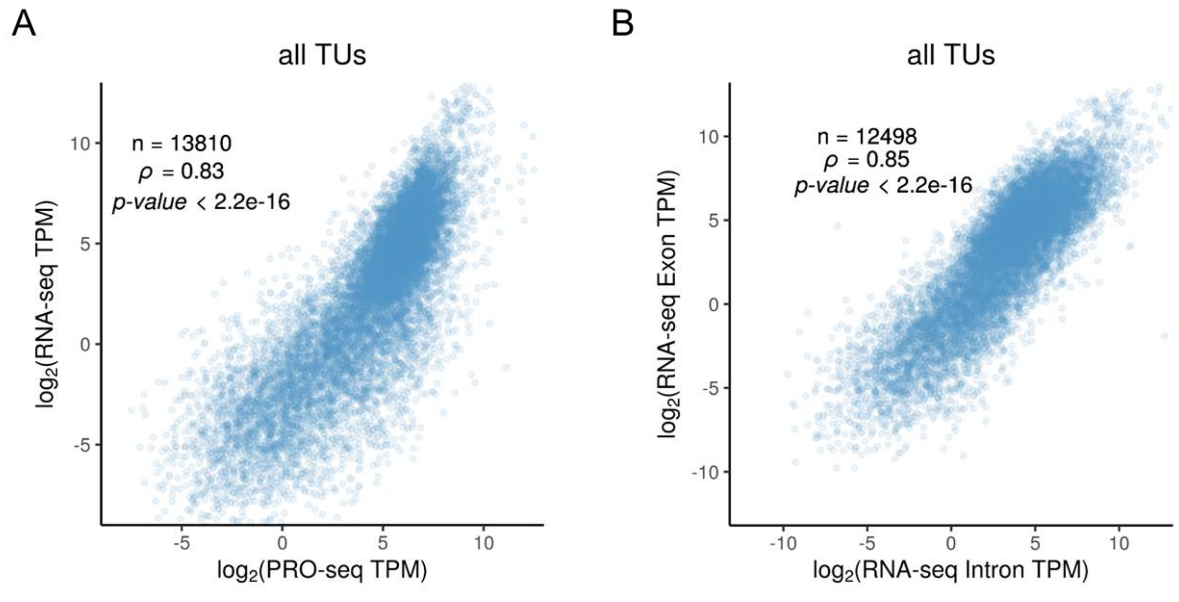

Supplemental Figure 2. Correlation of exonic reads from RNA-seq with either PRO-seq or intronic reads. (A) PRO-seq vs. exonic reads from RNA-seq. (B) Intronic reads vs. exonic reads from RNA-seq. Results are for all transcription units (TUs). ρ = Spearman's rank correlation coefficient.

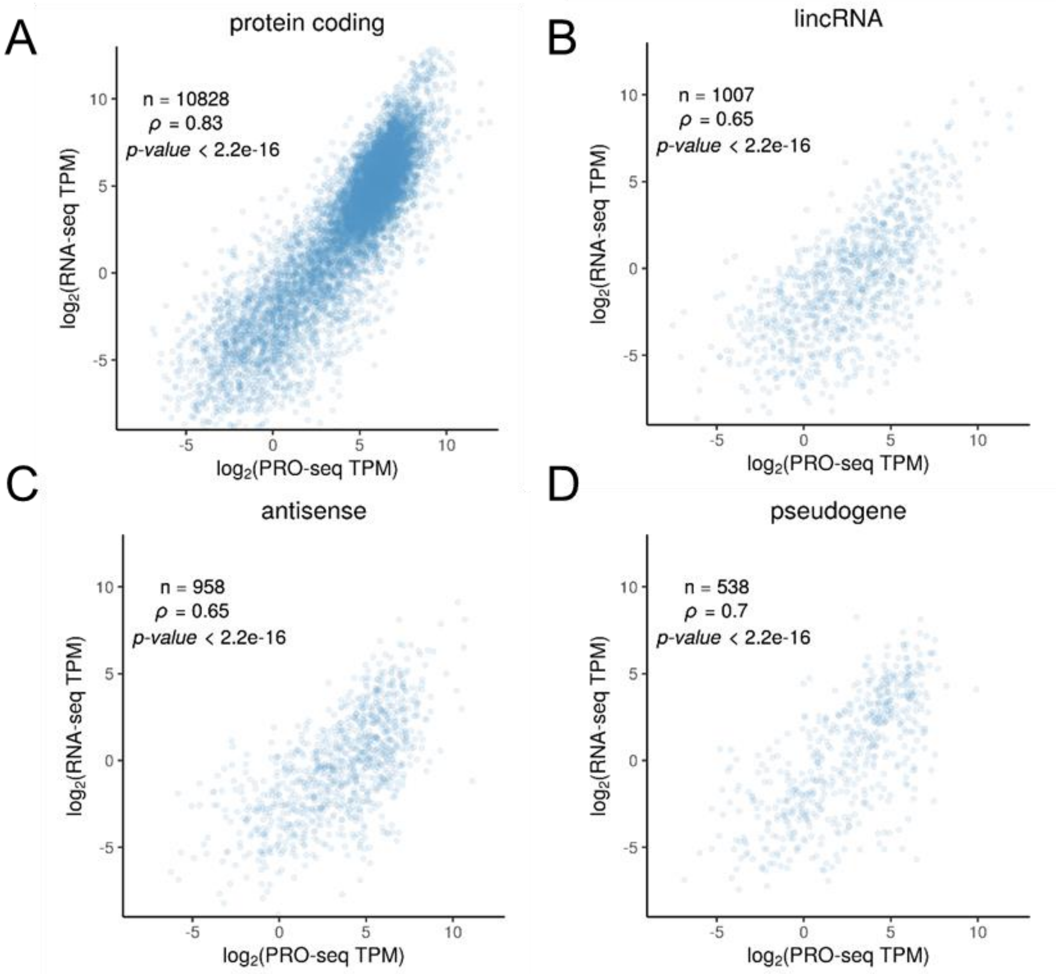

Supplemental Figure 3. Scatter plots of PRO-seq vs. RNA-seq for intron-containing transcription units in K562 cells. Panels describe (A) protein-coding mRNAs (B) intergenic lincRNAs (C) intragenic antisense non-coding genes, and (D) pseudogenes, all from GENCODE. For each plot, the number of genes analyzed is shown together with Spearman's rank correlation coefficient (ρ).

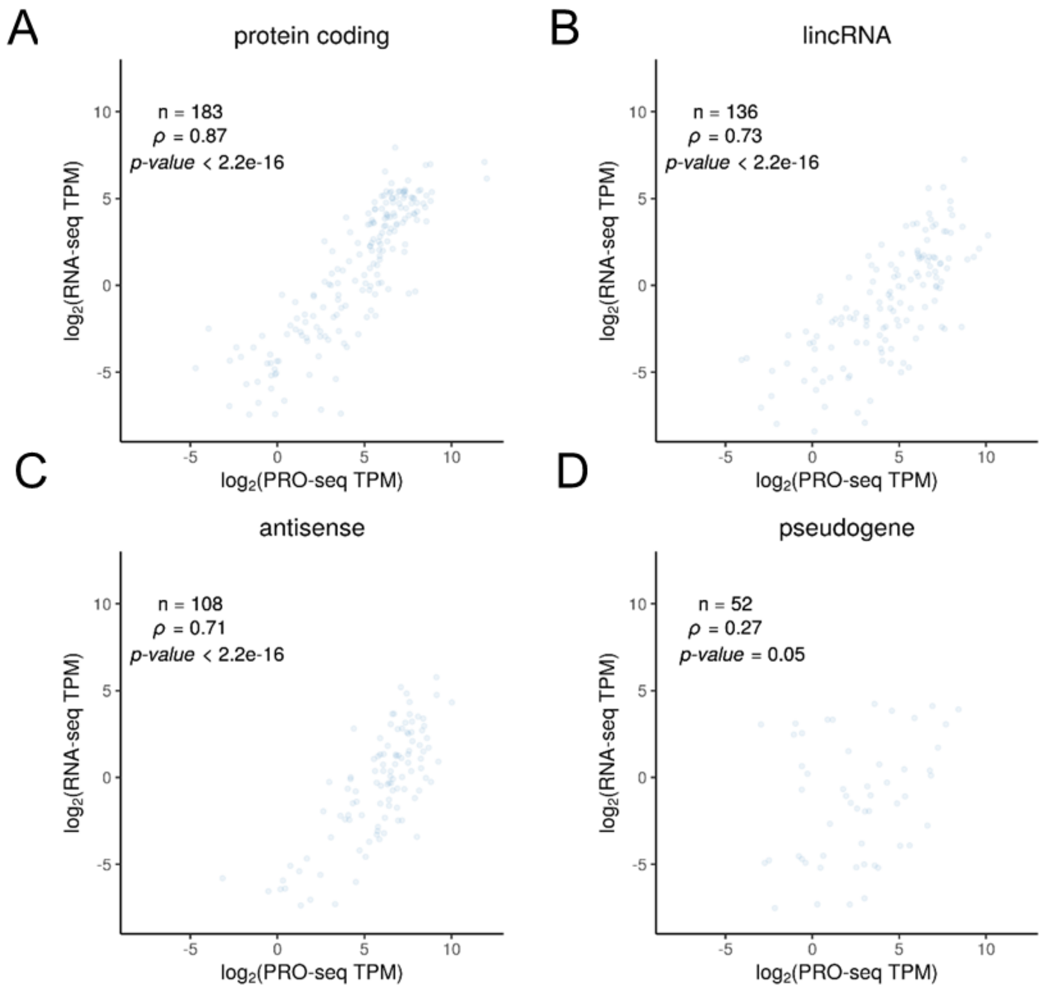

Supplemental Figure 4. Scatter plots of PRO-seq vs. RNA-seq for intron-less transcription units in K562 cells. Panels describe (A) protein-coding mRNAs (B) intergenic lincRNAs (C) intragenic antisense non-coding genes, and (D) pseudogenes, all from GENCODE. For each plot, the number of genes analyzed is shown together with Spearman's rank correlation coefficient (*ρ*).

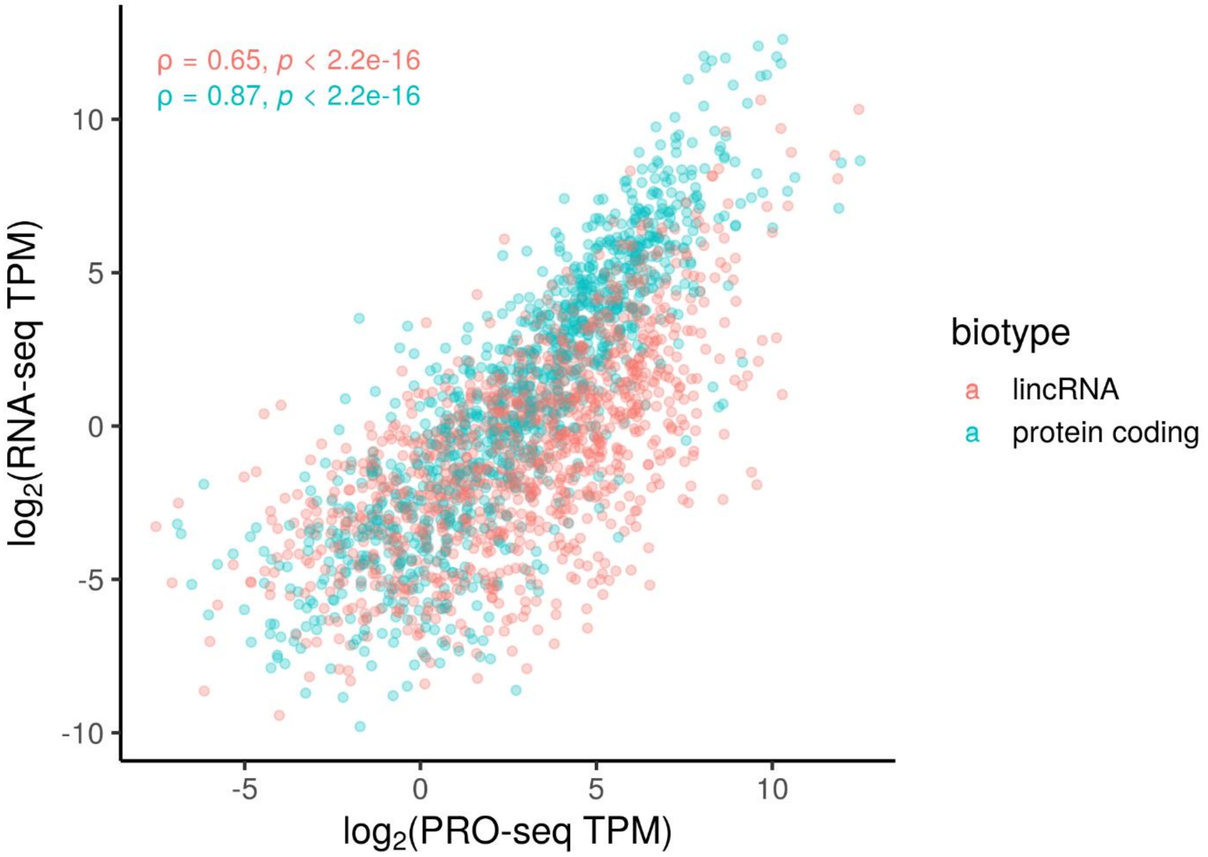

Supplemental Figure 5. Scatter plots of PRO-seq vs. RNA-seq for lincRNAs and protein-coding mRNAs in K562 cells after matching by PRO-seq signal. Subsampling was applied to obtain equal numbers of protein-coding mRNA and intergenic lincRNA (*n* = 1143) approximately matched by PRO-seq abundance (in TPM).

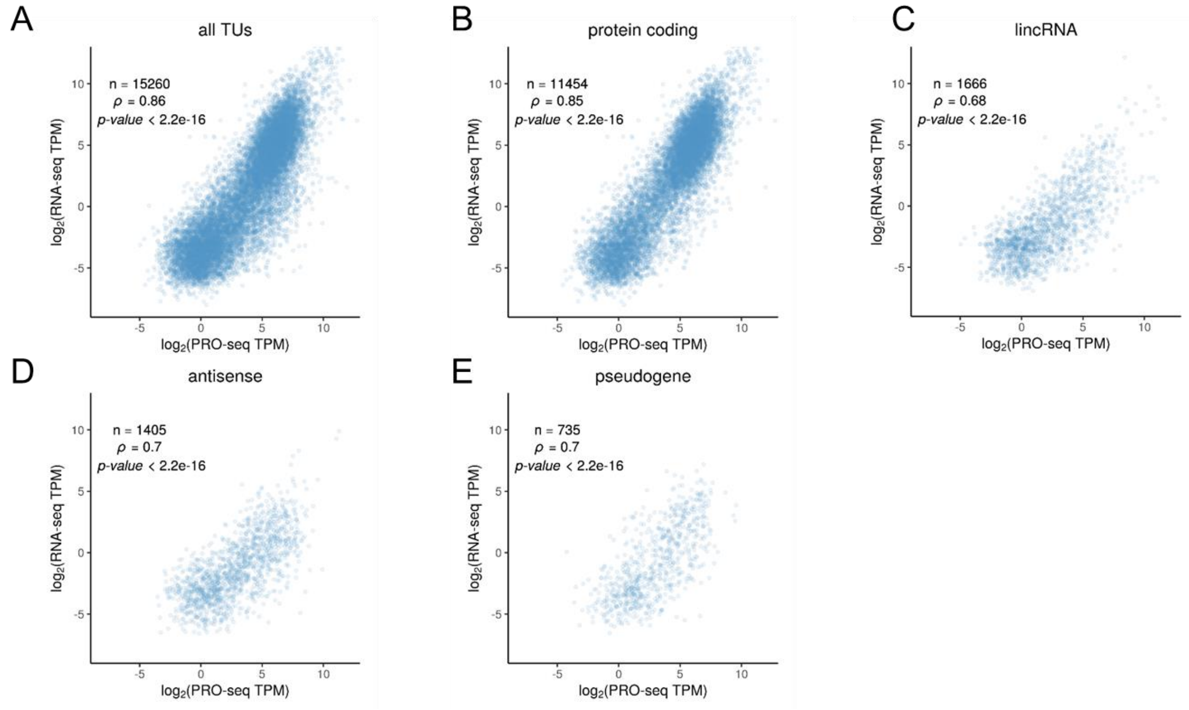

Supplemental Figure 6. Scatter plots of PRO-seq vs. RNA-seq for transcription units in K562 cells, based on newly collected data for this study. Panels describe (A) all annotated TUs, (B) protein-coding mRNAs, (C) intergenic lincRNAs, (D) intragenic antisense non-coding genes, and (E) pseudogenes. For each plot, the number of genes analyzed is shown together with Spearman's rank correlation coefficient (ρ).

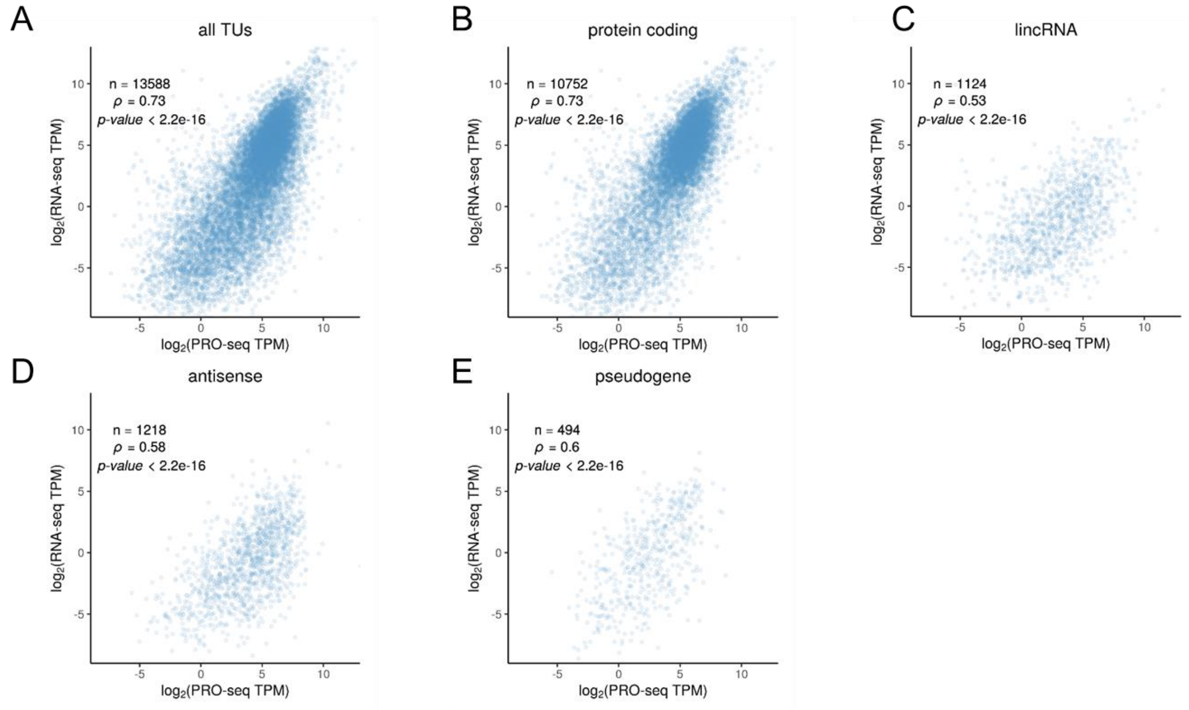

Supplemental Figure 7. Scatter plots of PRO-seq vs. RNA-seq for transcription units in HeLa cells. Panels describe (A) all annotated TUs, (B) protein-coding mRNAs, (C) intergenic lincRNAs, (D) intragenic antisense non-coding genes, and (E) pseudogenes. For each plot, the number of genes analyzed is shown together with Spearman's rank correlation coefficient (ρ).

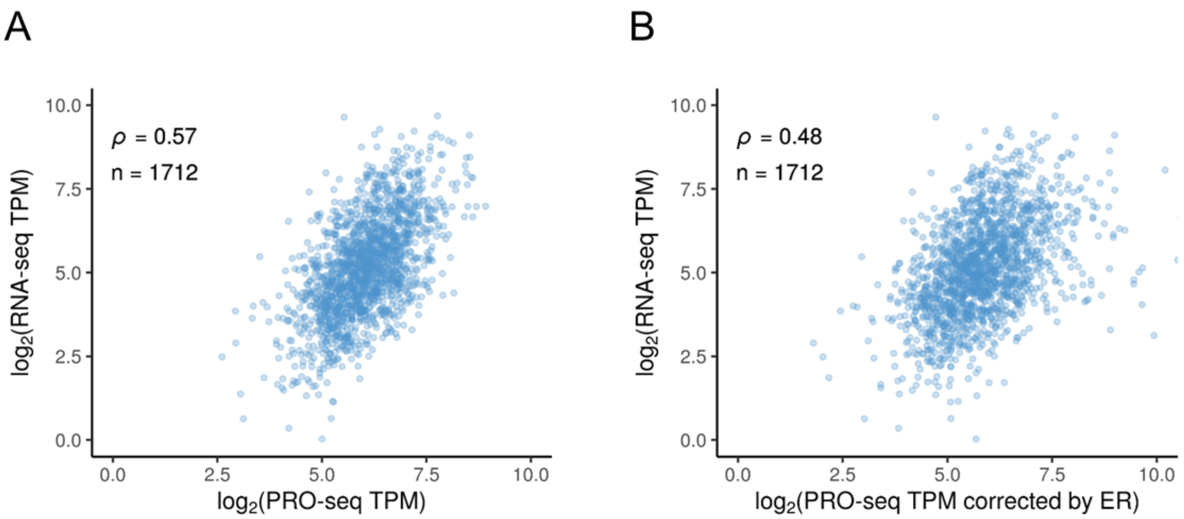

Supplemental Figure 8. Scatter plots of PRO-seq vs. RNA-seq for mRNAs and lincRNAs in K562 cells before and after correcting for elongation rate. (A) With uncorrected PRO-seq estimates. (B) With corrected PRO-seq estimates. Both plots describe *n*=1712 genes for which estimates of elongation rate were available from Veloso et. al.. Corrections in (B) were made by multiplying the PRO-seq TPM by the estimated elongation rate per gene (as described in the **Methods**).

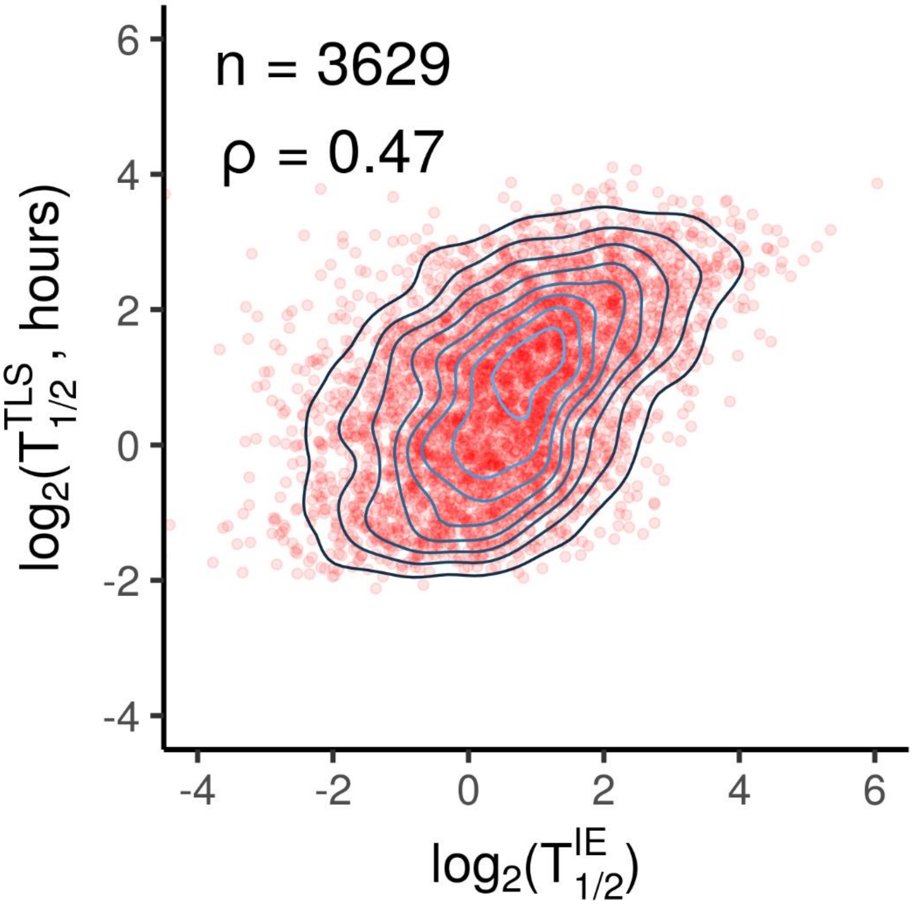

Supplemental Figure 9. Intronic half-life vs. TimeLapse-seq half-life. Scatter plot with density contours for $\log_2$(half-lives) estimated by the intronic/exonic RNA-seq method ($T_{1/2}^{IE}$, *x*-axis) vs. those estimated by TimeLapse-seq ($T_{1/2}^{TLS}$, *y*-axis) (n = 3629). ρ = Spearman's rank correlation coefficient.

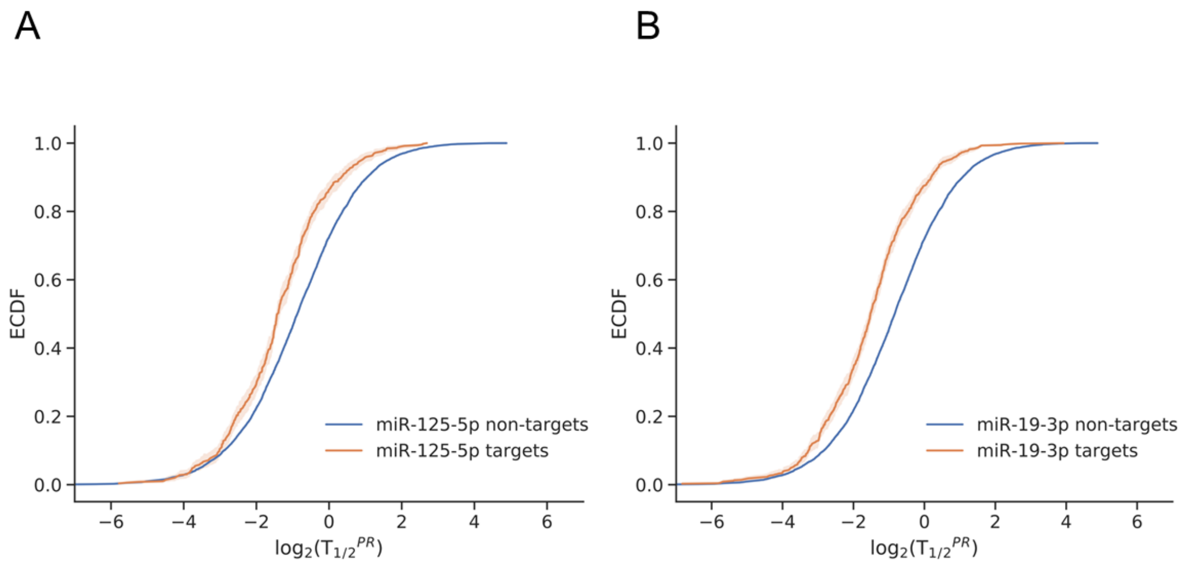

Supplemental Figure 10. Empirical cumulative distribution functions (eCDFs) for estimated half-lives of predicted targets of miR-125-5p and miR-19-3p vs. non-targets. The numbers of miR-125-5p targets and non-targets are 500 and 7662 (p = 5.09e-15, K-S test), respectively. The numbers of miR-19-3p targets and non-targets are 708 and 7454 (p = 1.49e-30, K-S test), respectively. Shading indicates 95% confidential intervals as estimated from 1000 bootstrap replicates.

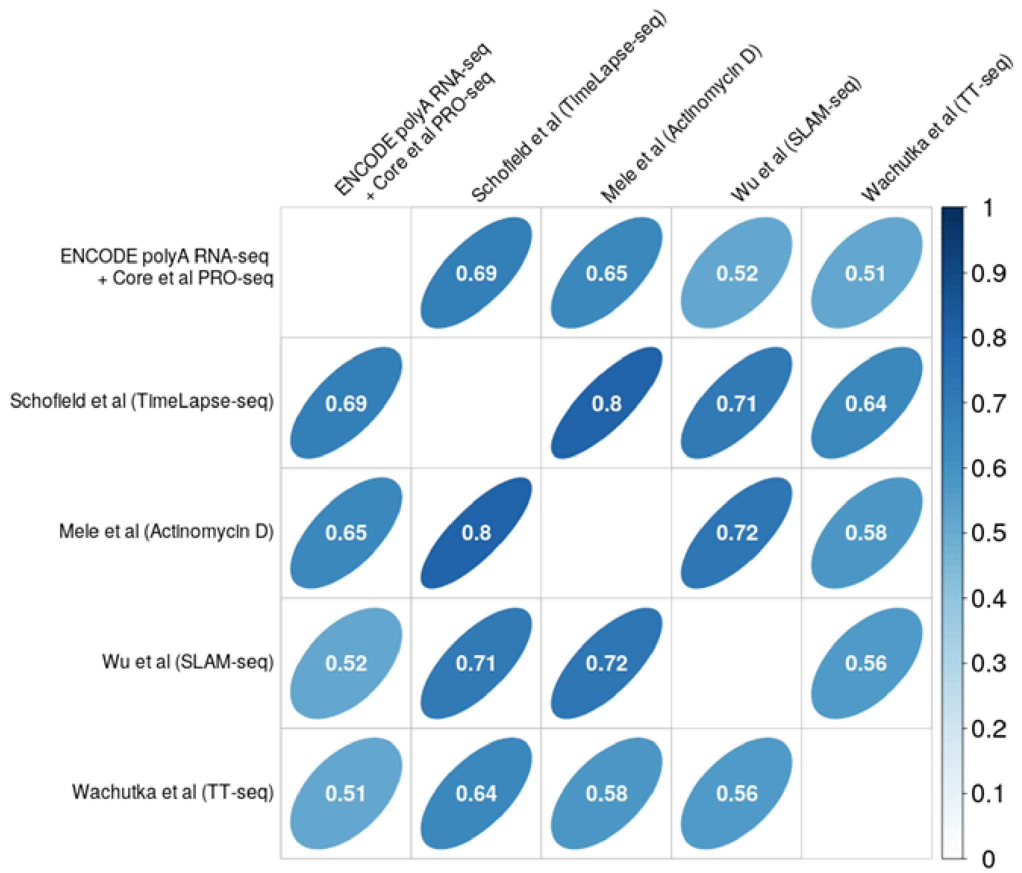

Supplemental Figure 11. Correlation of estimated RNA half-lives under various methods. Methods include our own, TimeLapse-seq, the method of Mele et al., SLAM-seq, and TT-seq. All comparisons are based on a common set of protein-coding genes to which all methods had been applied (n = 3449). The number in each cell of the matrix and the corresponding color represent Spearman's rank correlation coefficient (ρ) for each pairwise comparison.

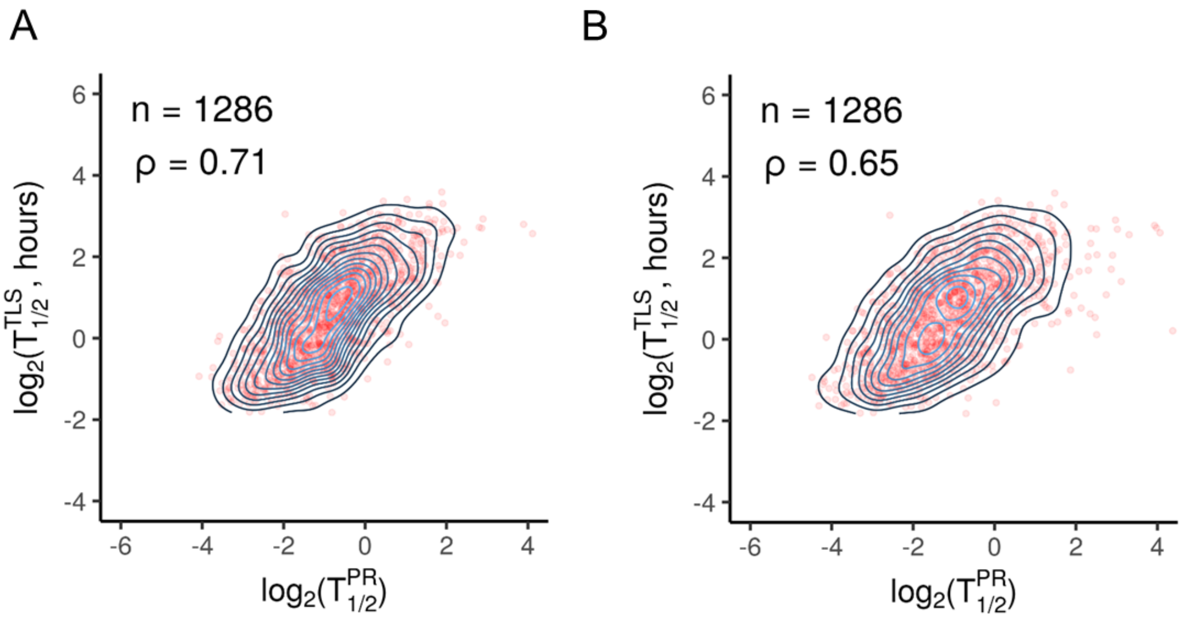

Supplemental Figure 12. Correlation of PRO-seq-based half-lives ($T_{1/2}^{PR}$, *x*-axis) vs. estimates from TimeLapse-seq ($T_{1/2}^{TLS}$, *y*-axis) after correcting for elongation rate. Elongation rates were obtained from Veloso et. al.. (A) half-life without correction (B) half-life with correction. The correction was performed as described in **Supplementary Fig. 8** and **Methods**. ρ = Spearman's rank correlation coefficient.

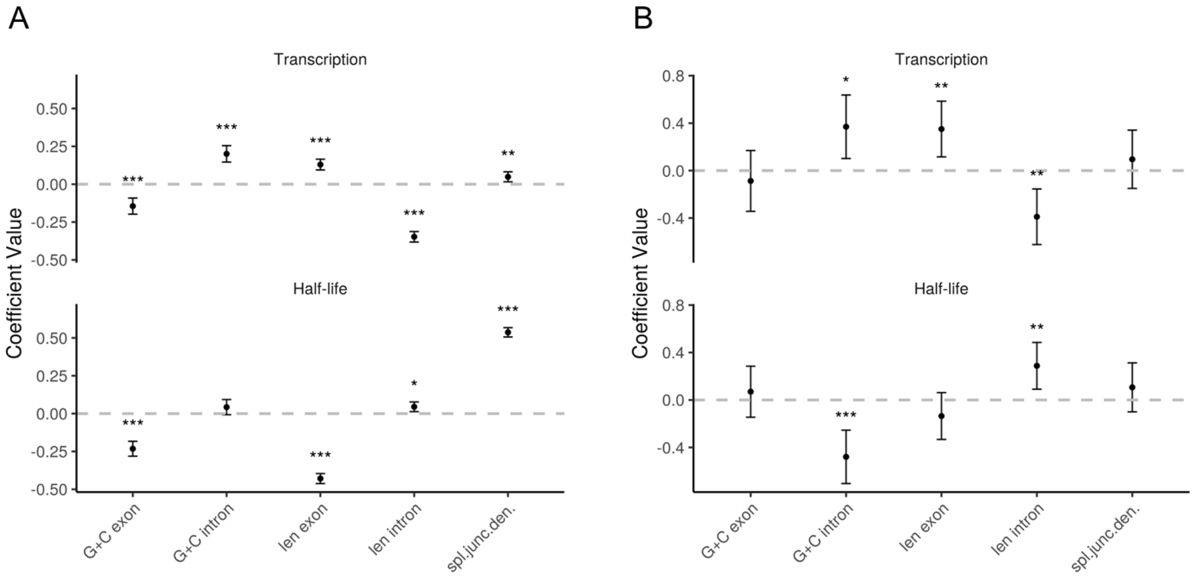

Supplemental Figure 13. SEM results for features of intron-containing transcription units in K562 cells. (A) Results for protein-coding mRNAs (*n* = 8428). (B) Results for lincRNA (*n* = 337). Features considered for each TU: G+C exon—GC content in exons; G+C Intron—G+C content in introns; len exon—Total exon length; len intron—Total intron length; spl. junc. den.—Number of splice junctions divided by mature RNA length. Error bars represent ±1.96 standard error, as calculated by the 'lavaan' R package**.** Significance (from *Z*-score): * $p<0.05$; ** $p<0.005$; *** $p<0.0005$.

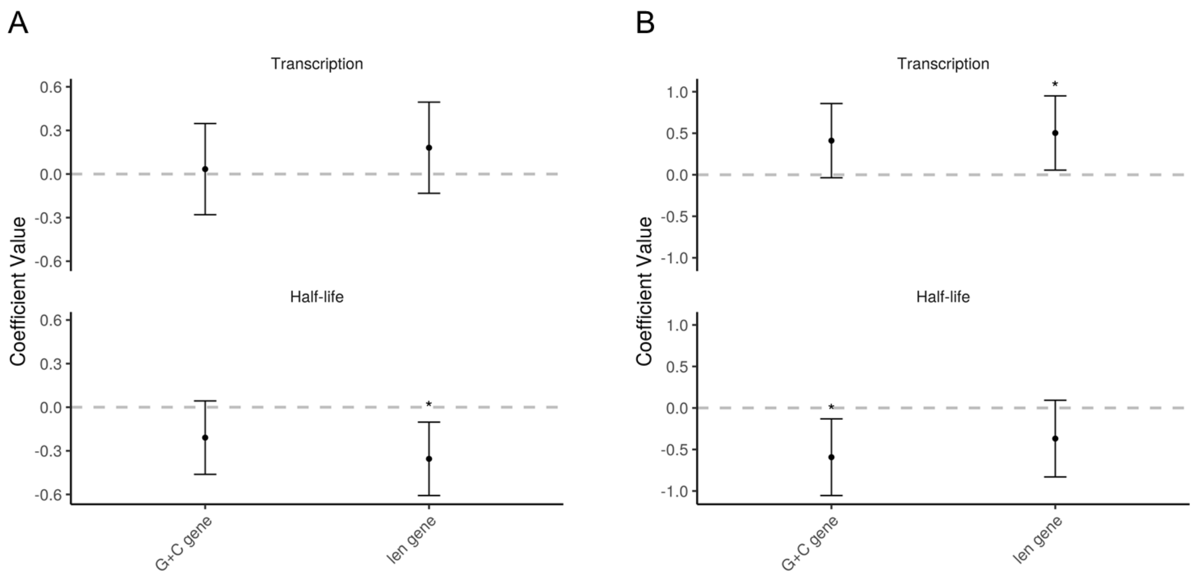

Supplemental Figure 14. SEM results for features of intron-less transcripts in K562 cells. (A) Results for protein-coding mRNAs (*n* = 108). (B) Results for lincRNAs (*n* = 55). Features considered for each TU: len gene—TU length; G+C gene—G+C content. Error bars represent ±1.96 standard error, as calculated by the 'lavaan' R package**.** Significance (from *Z*-score): * $p<0.05$; ** $p<0.005$; *** $p<0.0005$.

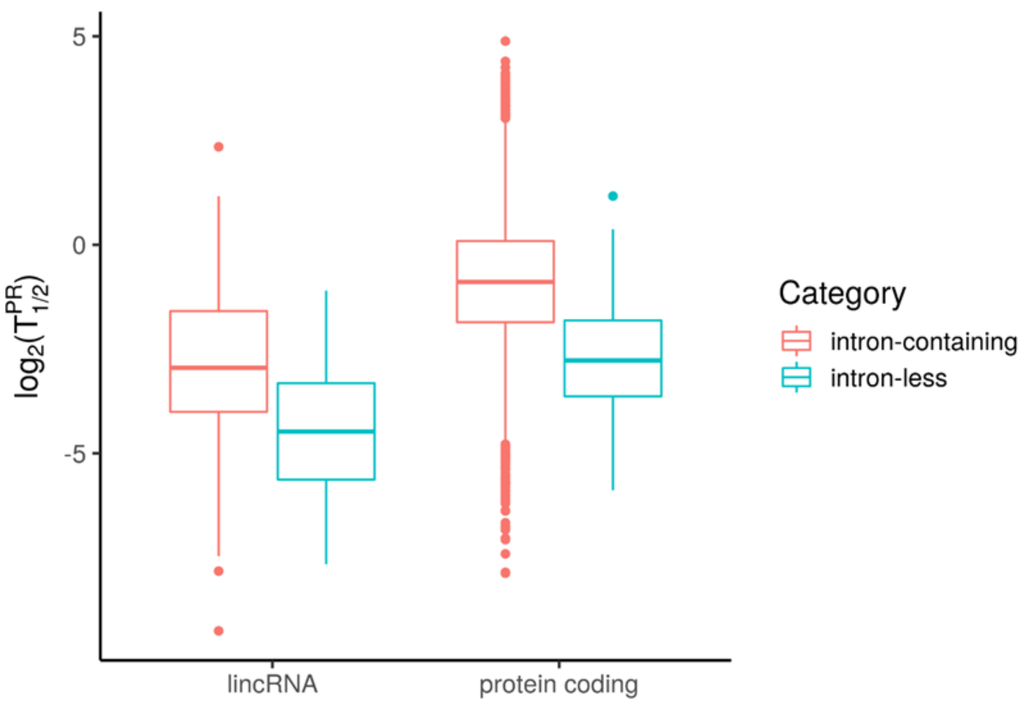

Supplemental Figure 15. Estimated half-lives for intron-containing and intron-less transcription units. Results for lincRNAs are shown on the *left* and results for protein-coding mRNAs are shown on the *right*.

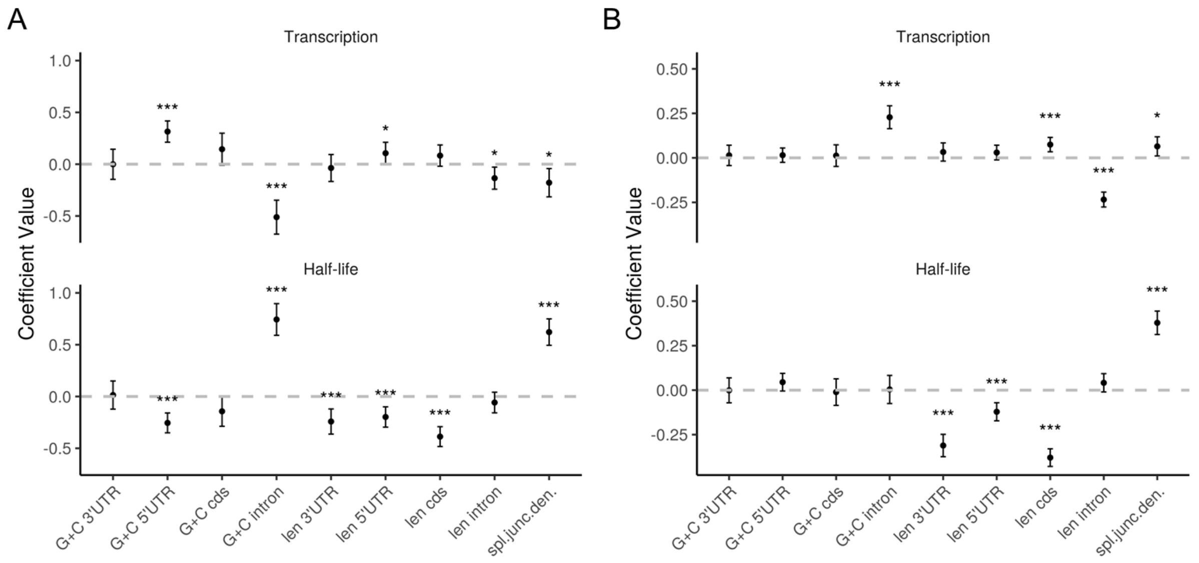

Supplemental Figure 16. SEM results for features of intron-containing transcripts in K562 cells, with and without a correction for elongation rate. (A) Results based on estimates of half-life that were explicitly corrected for elongation rates obtained from Veloso et al. as described in the **Methods**. (B) Results for the same set of genes without the correction for elongation rate. In both analyses, we used n = 1429 genes for which elongation-rate estimates were available.

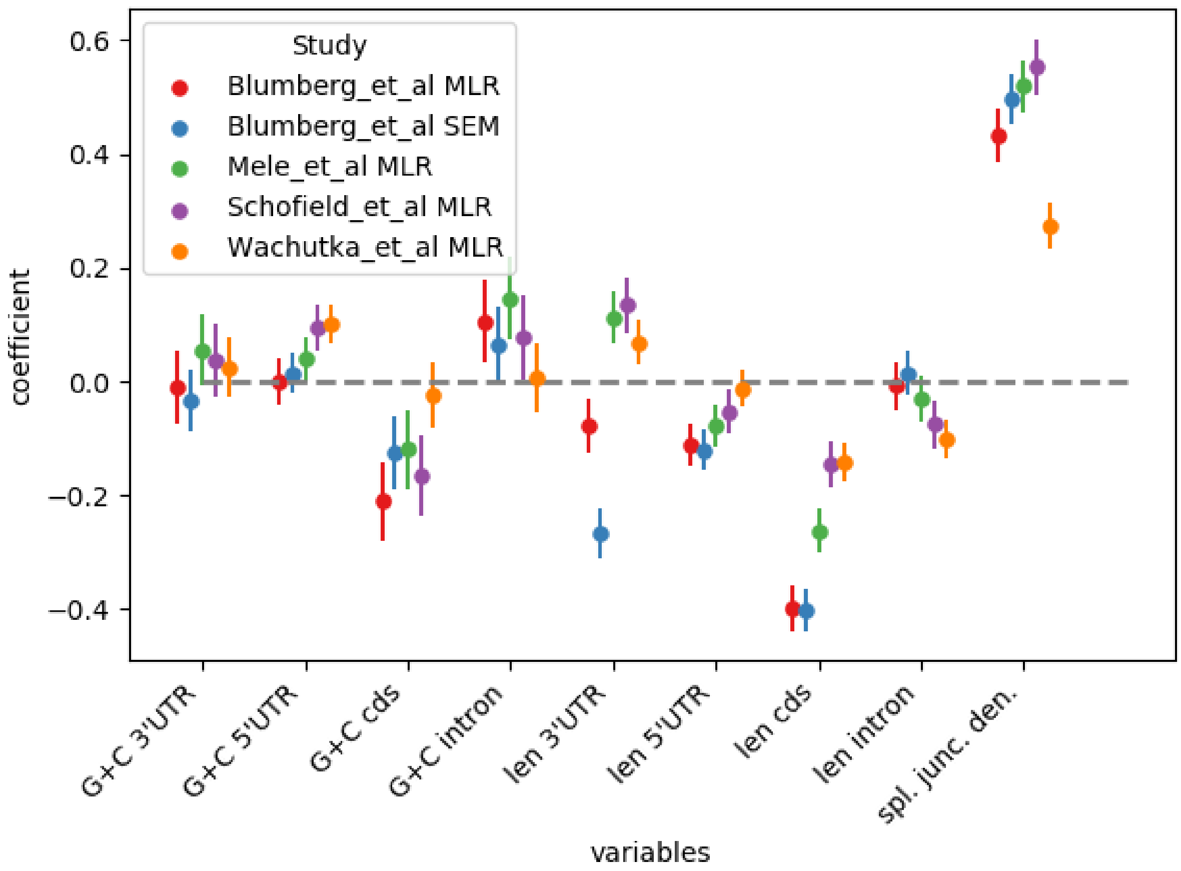

Supplemental Figure 17. Multiple linear regression (MLR) for features of transcription units versus RNA stability in K562 cells. The same analysis was repeated with half-life estimates from our study (Blumberg et al. MLR), the study of Mele et al., TimeLapse-seq (Schofield et al.), and TT-seq (Wachutka et al.). For comparison, we also show the coefficients from our Structural Equation Modeling analysis (Blumberg et al. SEM). Results are based on *n*=3418 genes to which all methods had been applied.

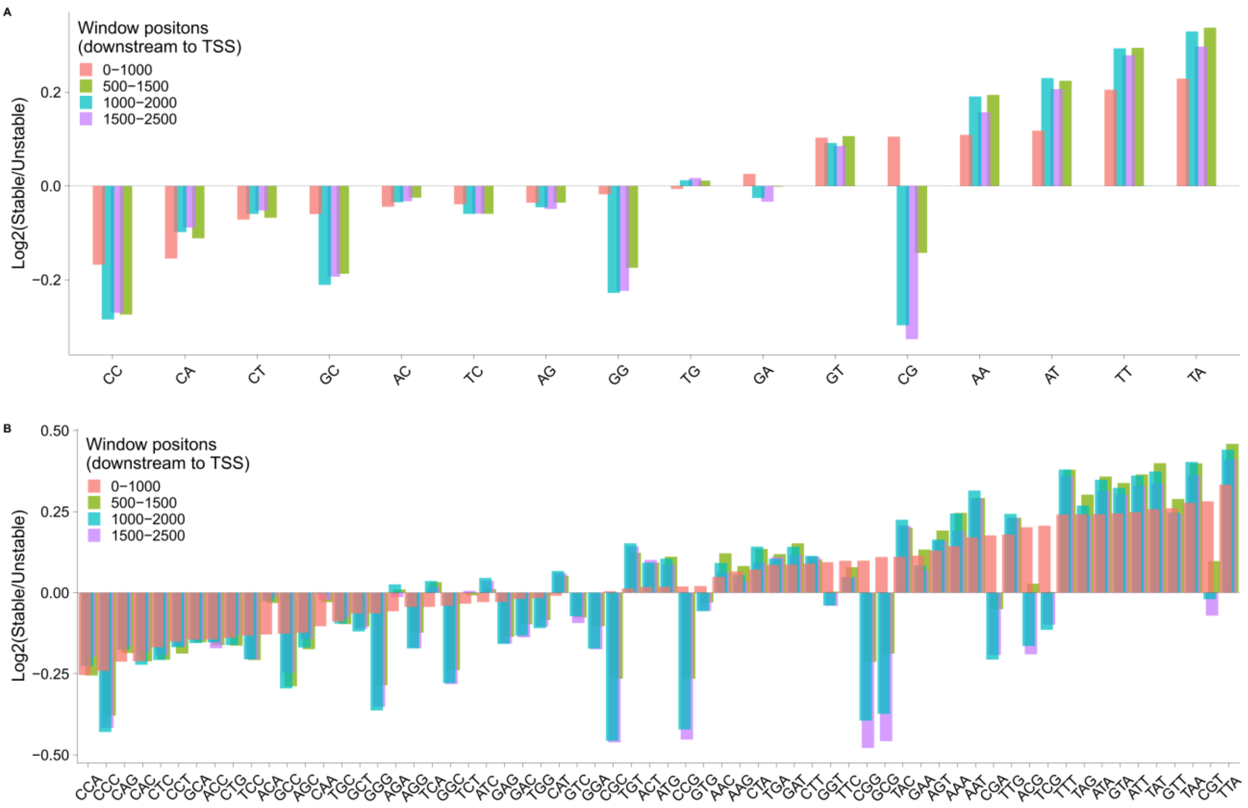

Supplemental Figure 18. DNA word enrichments in stable transcripts for protein-coding mRNAs. For each DNA word, we counted the numbers of total appearances of that word in all stable TUs (taking the top 20% by estimated half-life), $c_{s,w}$, and in all unstable transcripts (the bottom 20%), $c_{u,w}$. We then measured the stability enrichment as $\log_2(c_{s,w}/c_{u,w})$ (see **Methods**). The analysis was repeated in 1kb windows at four different distances downstream of the TSS (see legend). (A) Results for 2-mers. (B) Results for 3-mers. The order of the k-mers reflects the ($c_{s,w}/c_{u,w}$) ratio in the first window (0-1000bp).

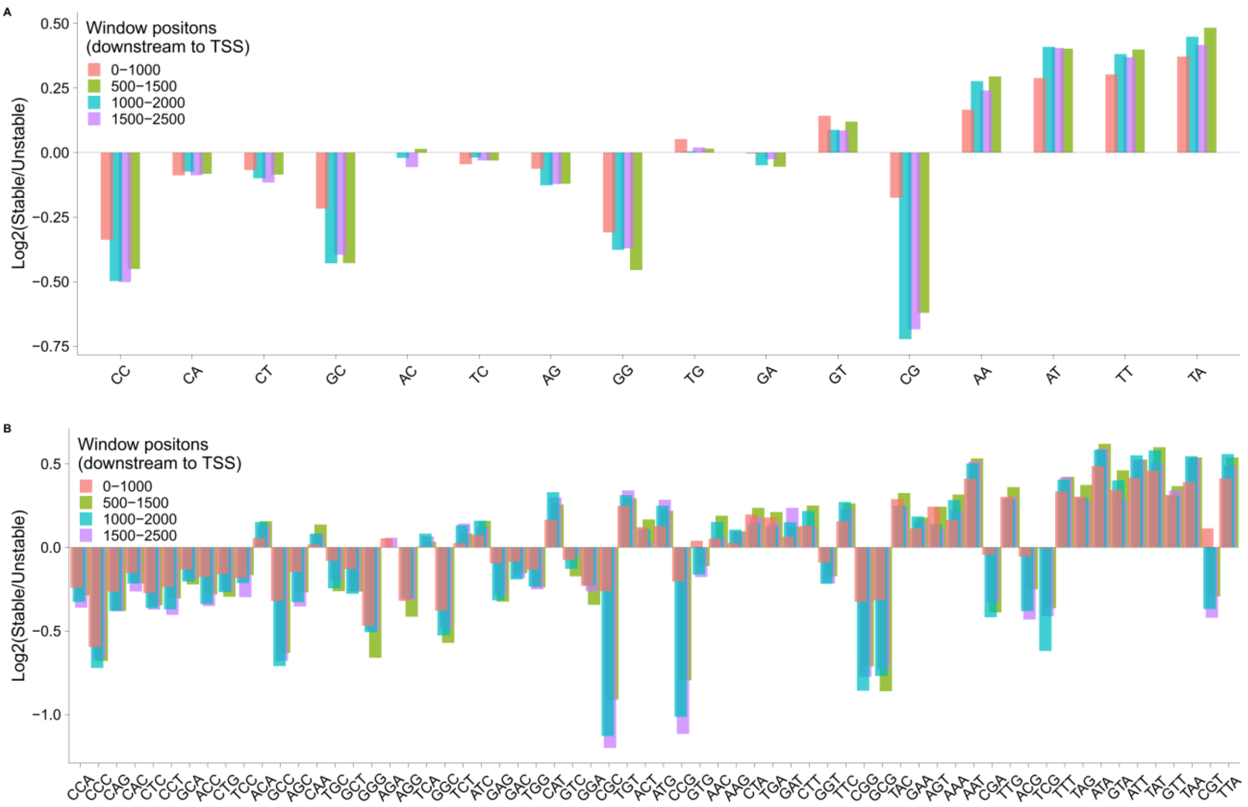

Supplemental Figure 19. DNA word enrichments in stable transcripts for lincRNAs. For each DNA word, we counted the numbers of total appearances of that word in all stable TUs (taking the top 20% by estimated half-life), $c_{s,w}$, and in all unstable transcripts (the bottom 20%), $c_{u,w}$. We then measured the stability enrichment as $\log_2(c_{s,w}/c_{u,w})$ (see **Methods**). The analysis was repeated in 1kb windows at four different distances downstream of the TSS (see legend). (A) Results for 2-mers. (B) Results for 3-mers. The order of the k-mers reflects the ($c_{s,w}/c_{u,w}$) ratio in the first mRNA window (0-1000bp; see **Supplemental Fig. 18**).

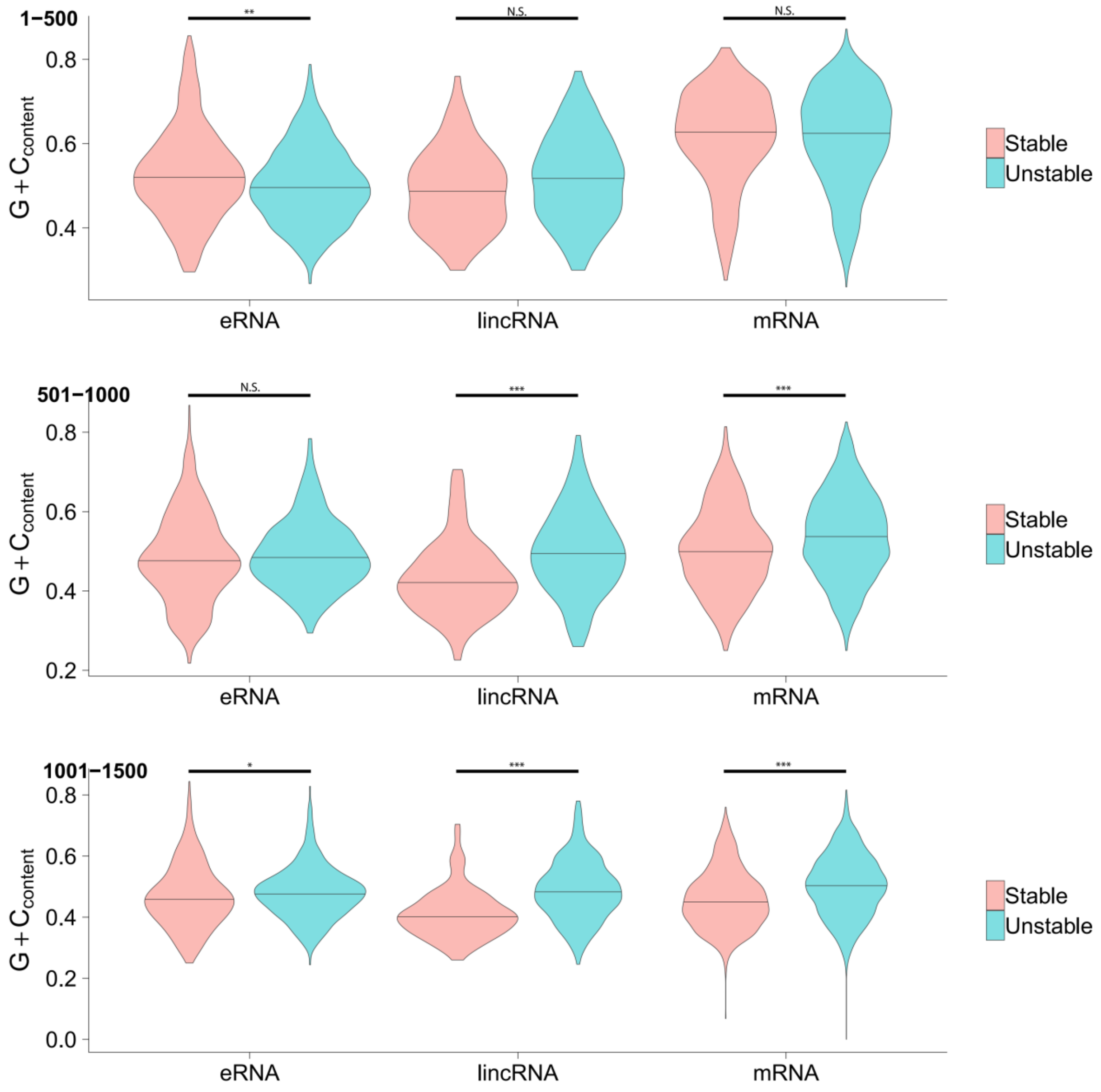

Supplemental Figure 20. **G+C content in intervals downstream of the TSS for various classes of transcription units**. Results are shown for intervals 1-500bp (*top*), 501-1000bp (*middle*), and 1001-1500bp (*bottom*) downstream of the TSS. * p<0.01; ** p<0.001; *** p<0.0001, Mann–Whitney U test. enhancer RNAs (eRNA, stable: n=510; unstable: n=510), lincRNAs (stable: n=91; unstable: n=198) and mRNAs (stable: n=919; unstable: n=2146).

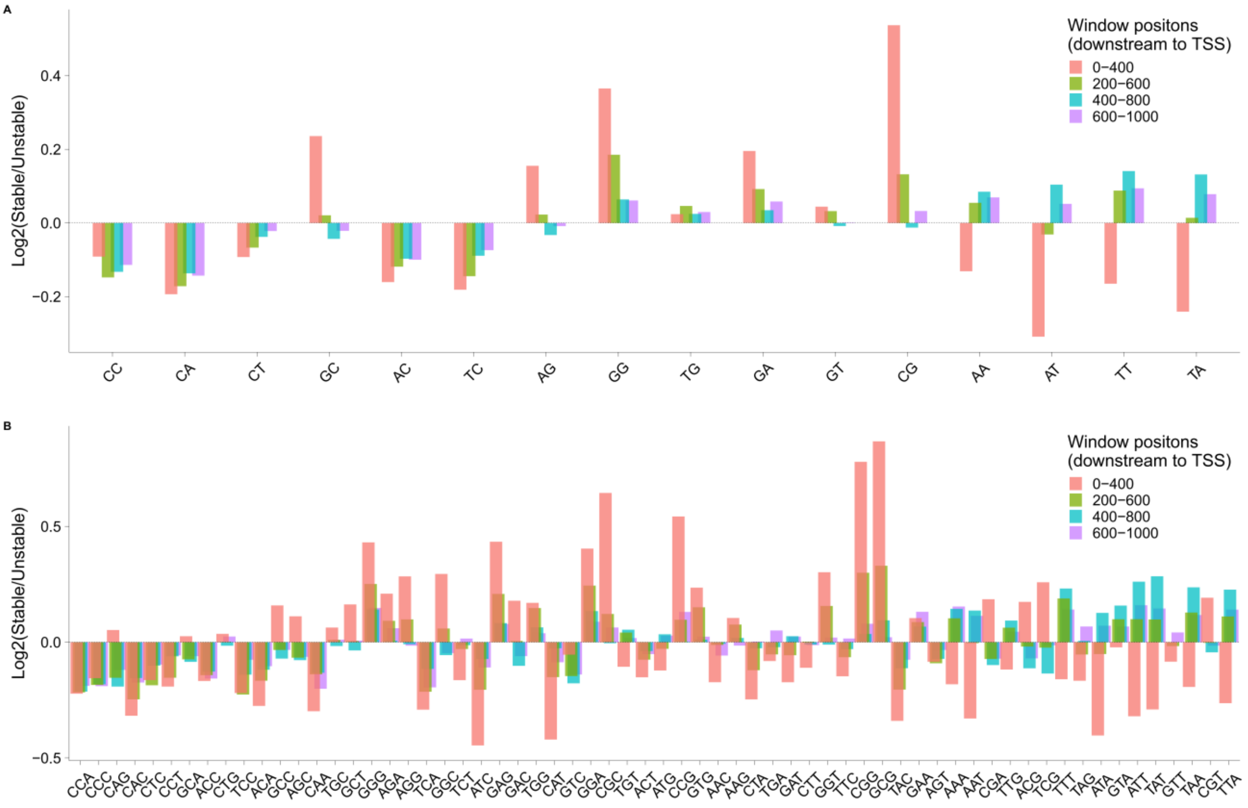

Supplemental Figure 21. DNA word enrichments in stable transcripts for eRNAs. For each DNA word, we counted the numbers of total appearances of that word in all stable TUs (taking the top 20% by estimated half-life), $c_{s,w}$, and in all unstable transcripts (the bottom 20%), $c_{u,w}$. We then measured the stability enrichment as $\log_2(c_{s,w}/c_{u,w})$ (see **Methods**). The analysis was repeated in 400bp windows at four different distances downstream of the TSS (see legend). (A) Results for 2-mers. (B) Results for 3-mers. The order of the k-mers reflects the $(c_{s,w}/c_{u,w})$ ratio in the first mRNA window (0-1000bp; see **Supplemental Fig. 18**).

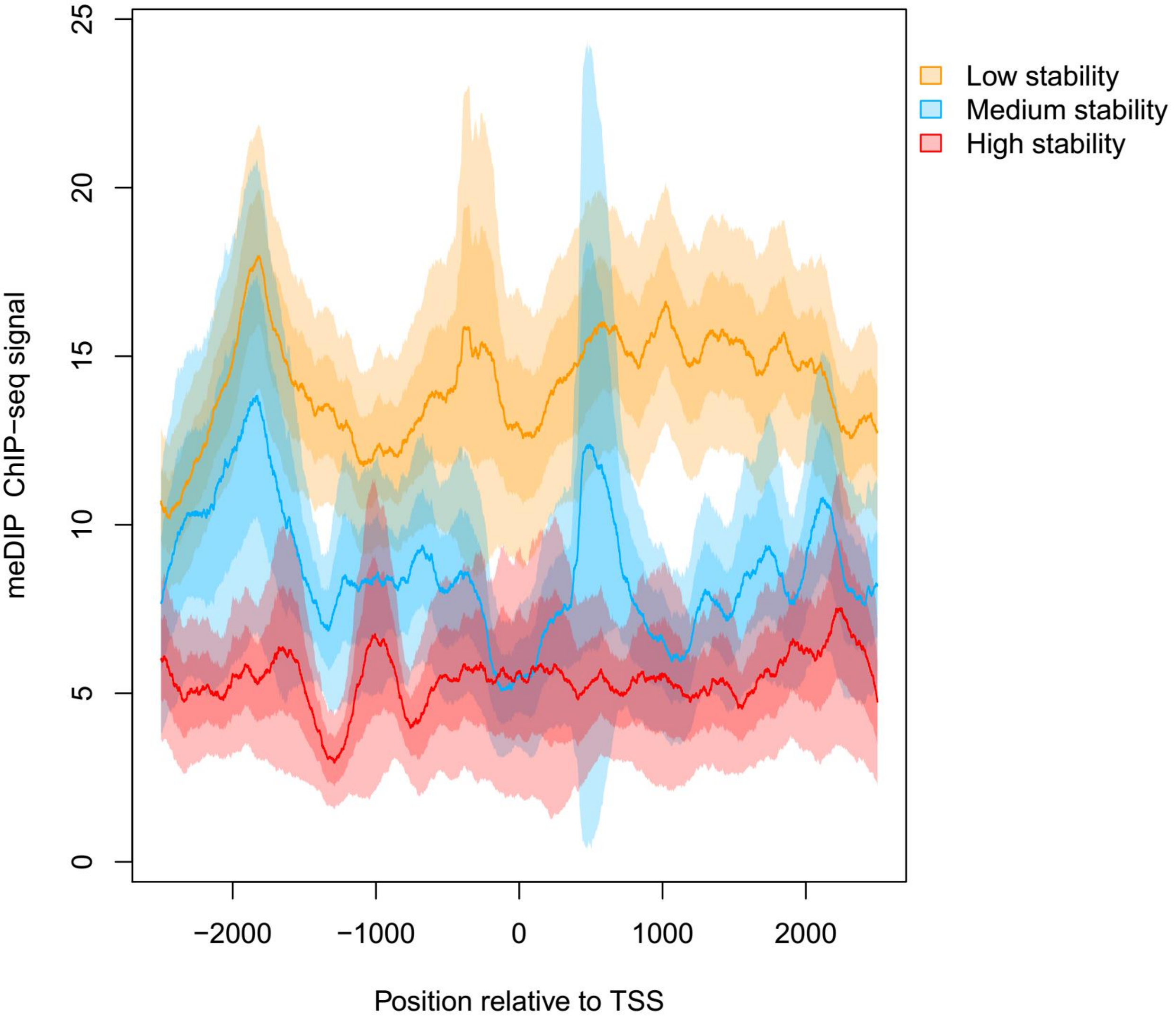

Supplemental Figure 22. DNA methylation in lincRNAs of various stability levels. Plots represent the average signal of the methylated DNA immunoprecipitation (MeDIP-seq) assay in K562 cells in three sets of lincRNAs: Low stability (lowest 20% by estimated half-life); Medium stability (40%-60%); and High stability (highest 20%).

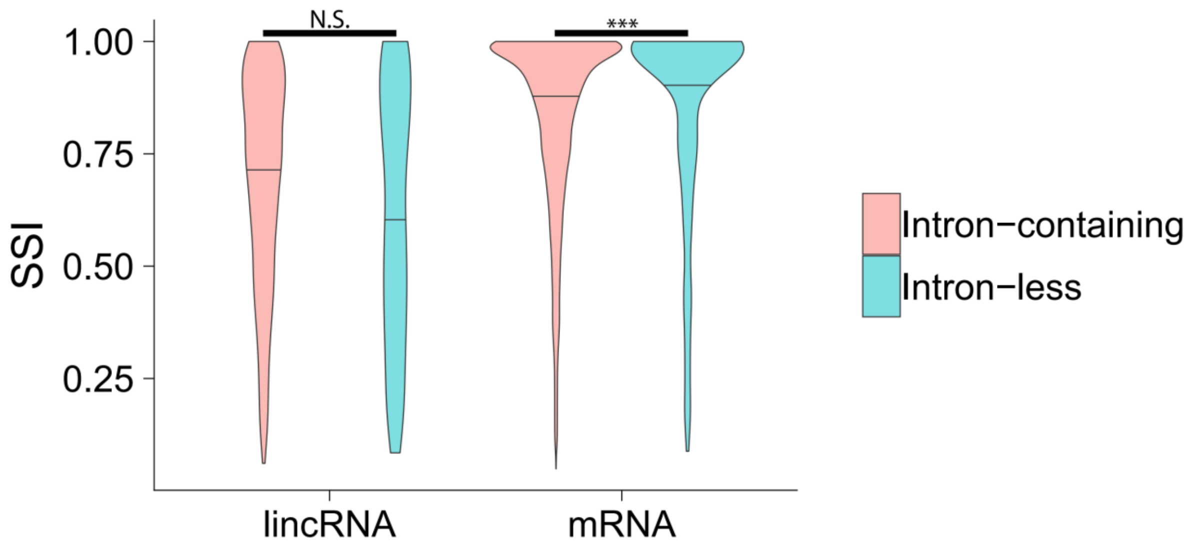

Supplemental Figure 23. Sequence Stability Index of intron-containing versus intron-less genes. Results are shown for protein-coding mRNAs (intron-containing: *n*=10728; intron-less: *n*=179) and lincRNAs (intron-containing: *n*=989; intron-less: *n*=136). * p<0.01; ** p<0.001; *** p<0.0001, Mann–Whitney U test.

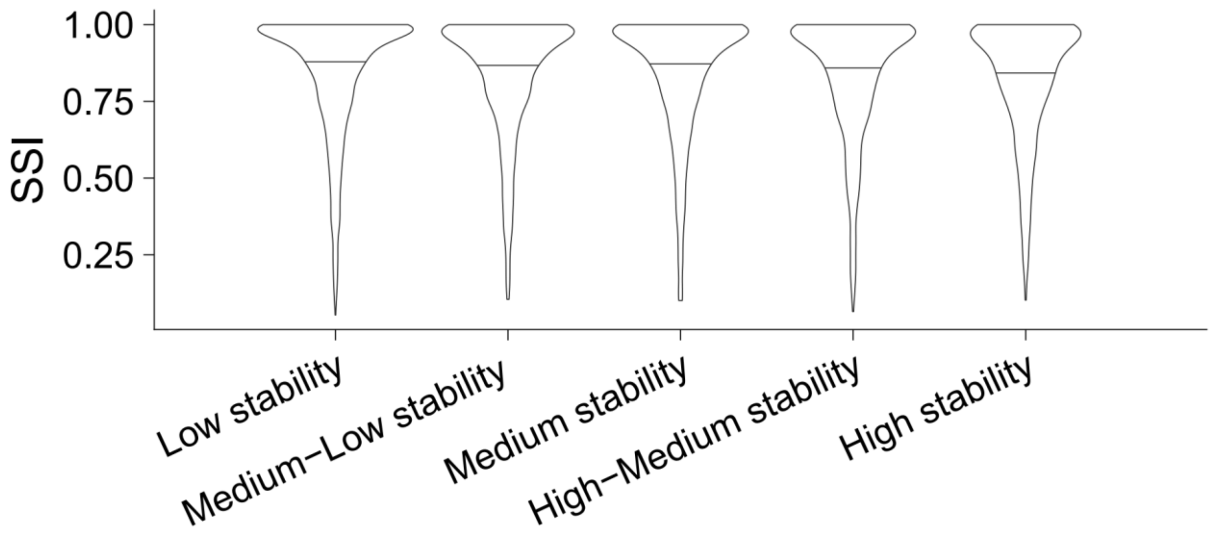

Supplemental Figure 24. Sequence Stability Index (SSI) for mRNAs of various stability classes. Each category represents a decile ranging from lowest stability to highest stability, based on the estimated half-lives.

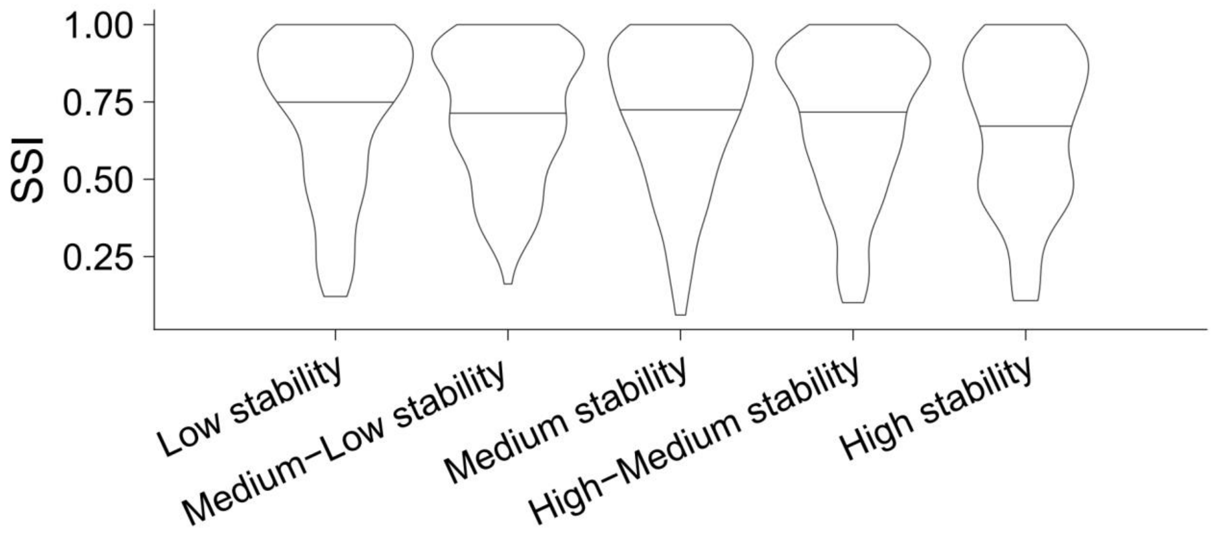

Supplemental Figure 25. Sequence Stability Index (SSI) for lincRNAs of various stability classes. Each category represents a quintile ranging from lowest stability to highest stability, based on the estimated half-lives.

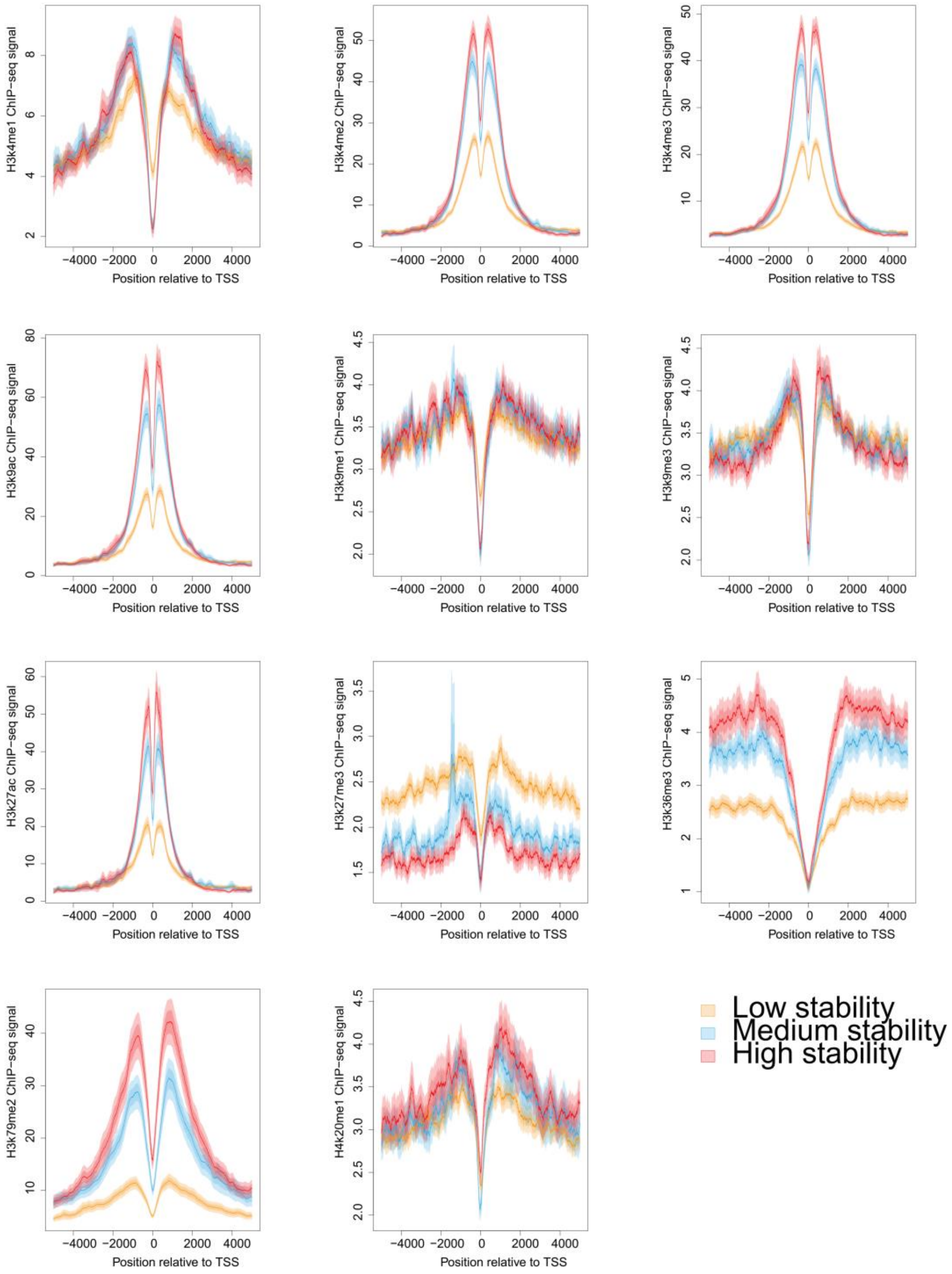

Supplemental Figure 26. **Histone modification signals for protein-coding mRNAs of various stability classes.** 11 different histone-modifications are shown. In each panel, separate plots are shown for mRNAs in three stability classes: Low stability (lowest 20% by estimated half-life); Medium stability (40%-60%); and High stability (highest 20%). These results are based on the previously published data (see **Methods**); see **Supplementary Figure 27** for similar results based on newly collected PRO-seq and RNA-seq data.

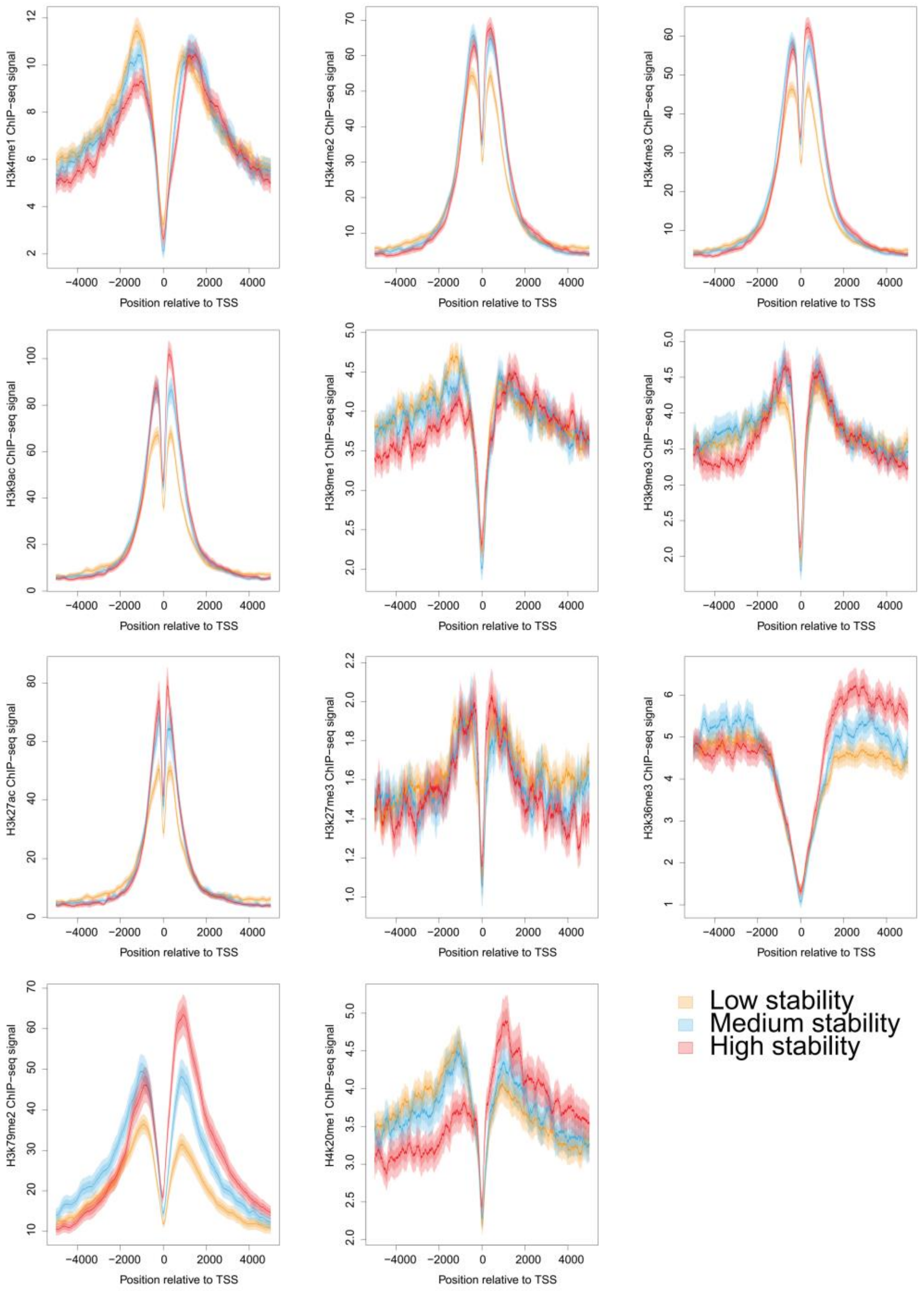

Supplemental Figure 27. **Histone modification signals for protein-coding mRNAs of various stability classes, based on newly collected data for this study (cf. Supplementary Figure 26).** 11 different histone-modifications are shown. In each panel, separate plots are shown for mRNAs in three stability classes: Low stability (lowest 20% by estimated half-life); Medium stability (40%-60%); and High stability (highest 20%).

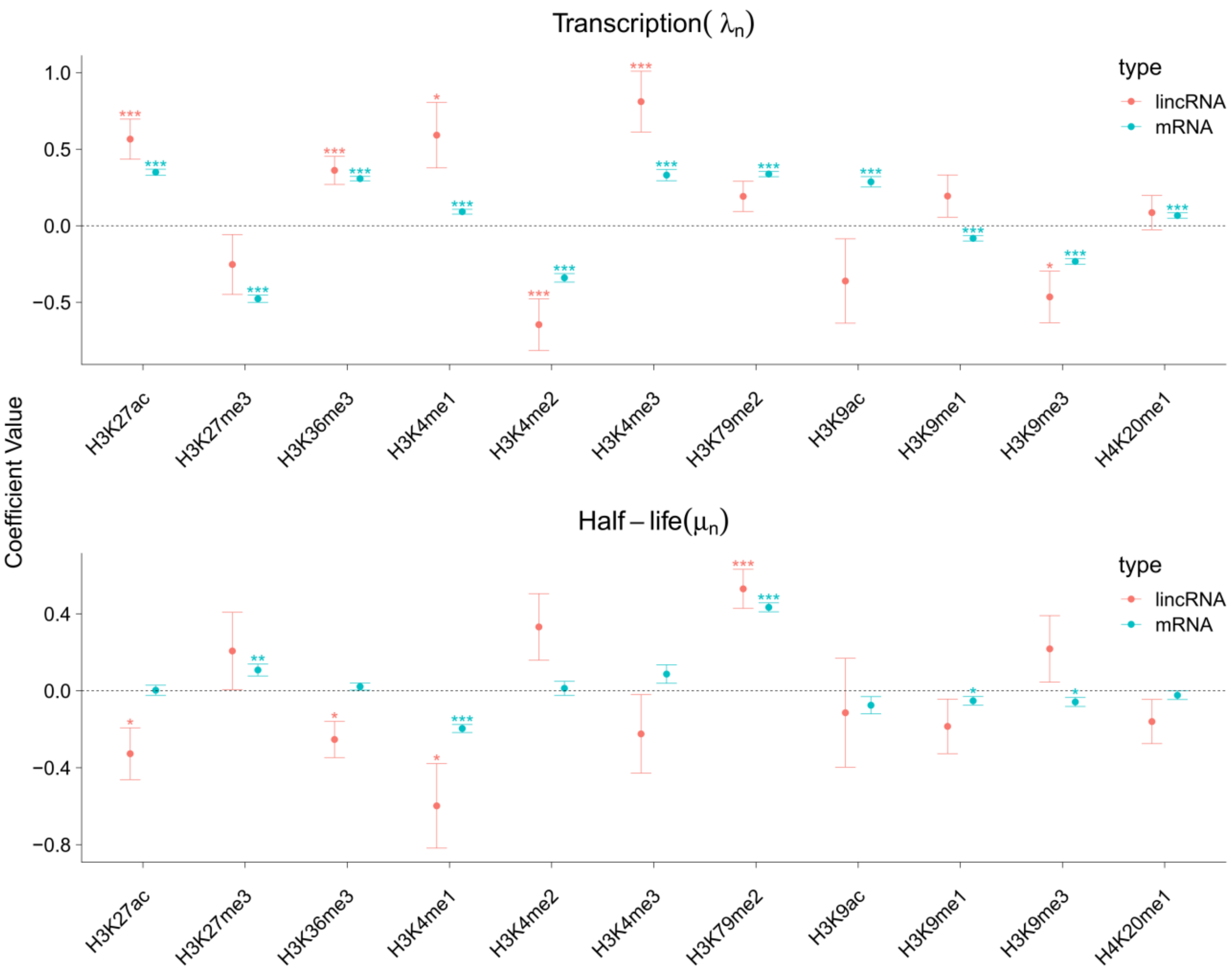

Supplemental Figure 28. Estimated SEM coefficients for transcription (*λ_n*; top) and half-life (*μ_n*; bottom) for 11 histone modifications. It's assayed by ChIP-seq in the 500 bases immediately downstream of the TSS. The entire set of genes of each type was analyzed. Error bars and significance are as in **Fig. 3B**

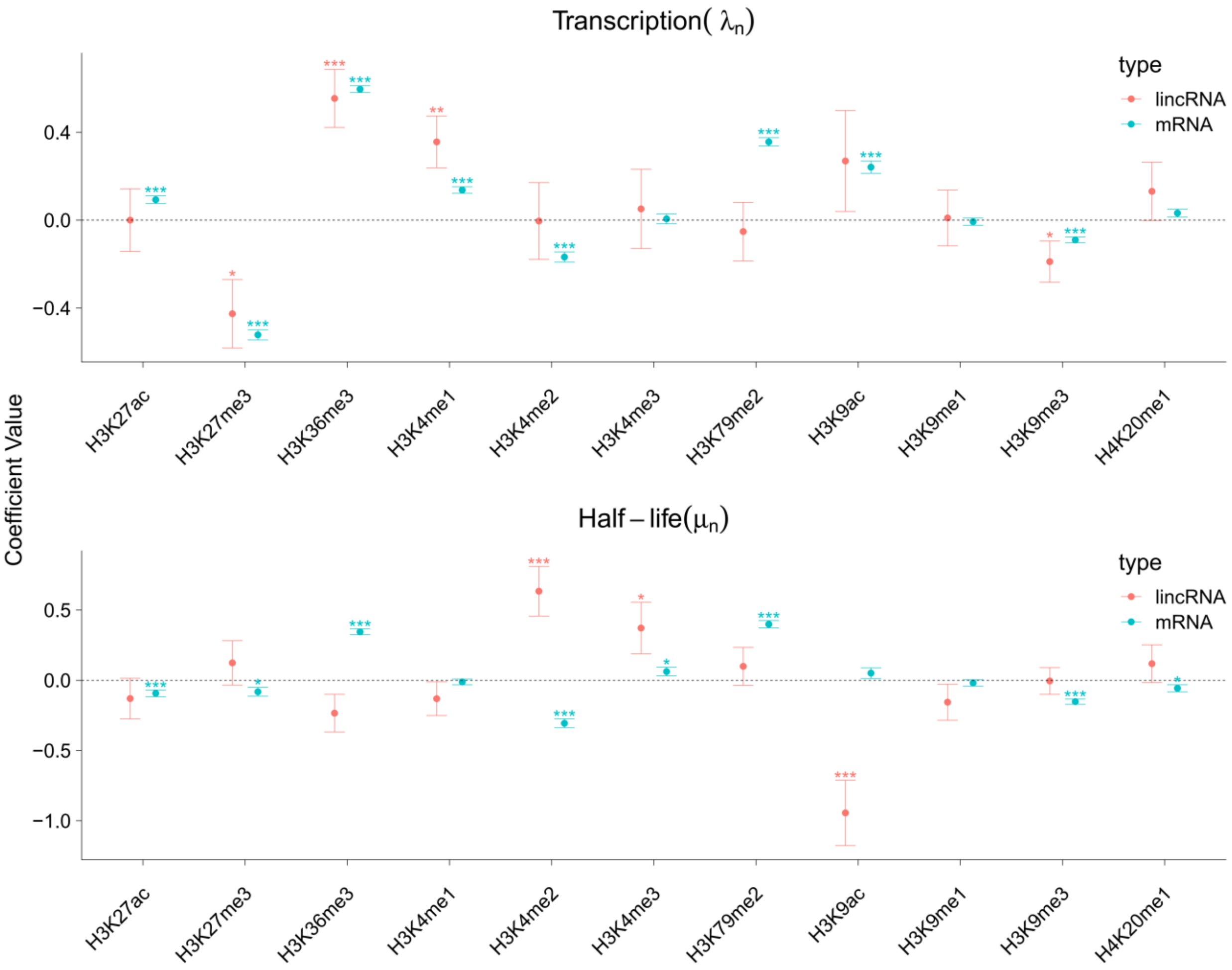

Supplemental Figure 29. Estimated SEM coefficients for transcription (*λ_n*; top) and half-life (*μ_n*; bottom) for 11 histone modifications. It's assayed by ChIP-seq in the 1000-1500 bases downstream of the TSS. The entire set of genes of each type was analyzed. Error bars and significance are as in **Fig. 3B**

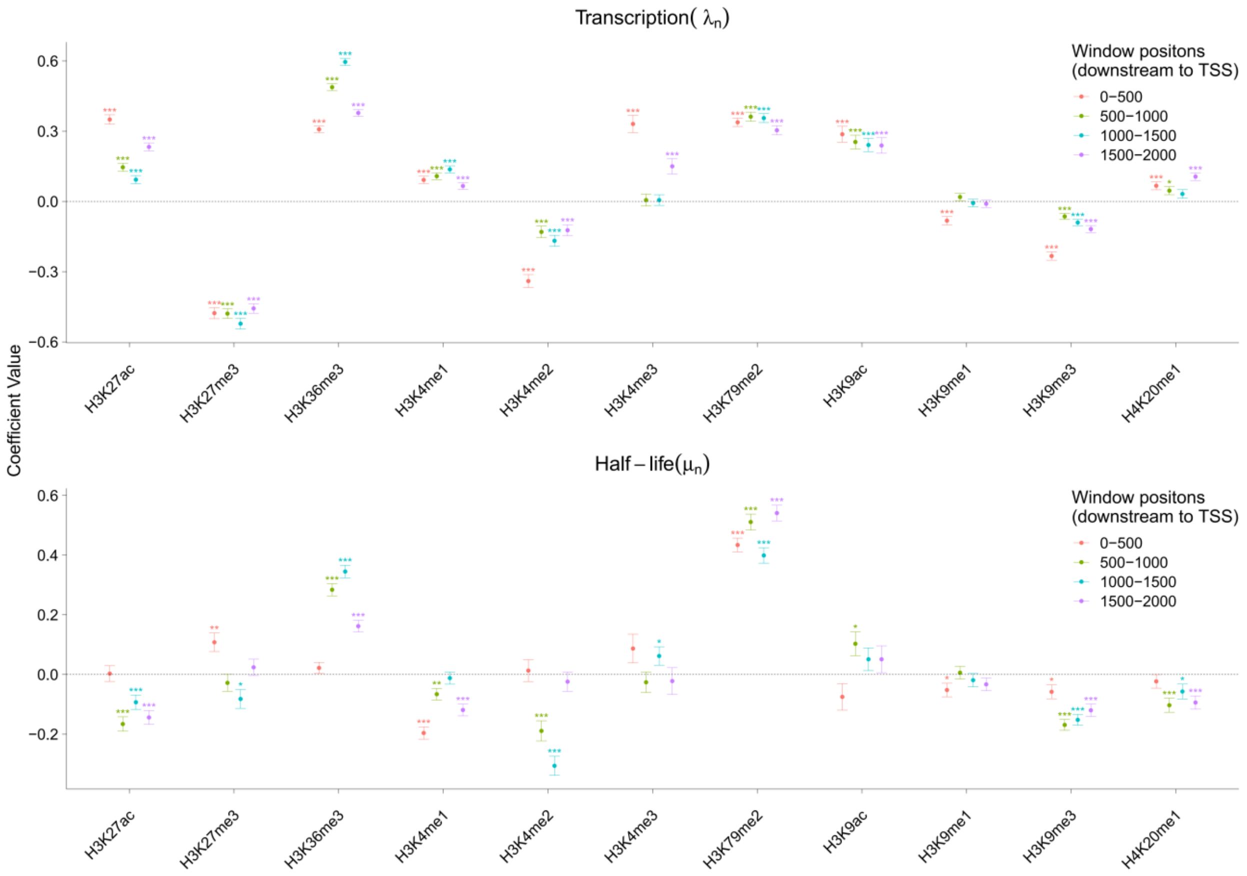

Supplemental Figure 30. Estimated SEM coefficients for transcription (λ_n; top) and half-life (μ_n; bottom) for 11 histone modifications. It's assayed by ChIP-seq in 4 windows, each of 500 bases downstream of the TSS (0-500, 500-1000, 1000-1500, 1500-2000, represented by colors). The entire set of mRNA genes was analyzed. Error bars and significance are as in **Fig. 3B.** These results are based on the previously published data (see **Methods**); see **Supplementary Figure 31** for similar results based on newly collected PRO-seq and RNA-seq data.

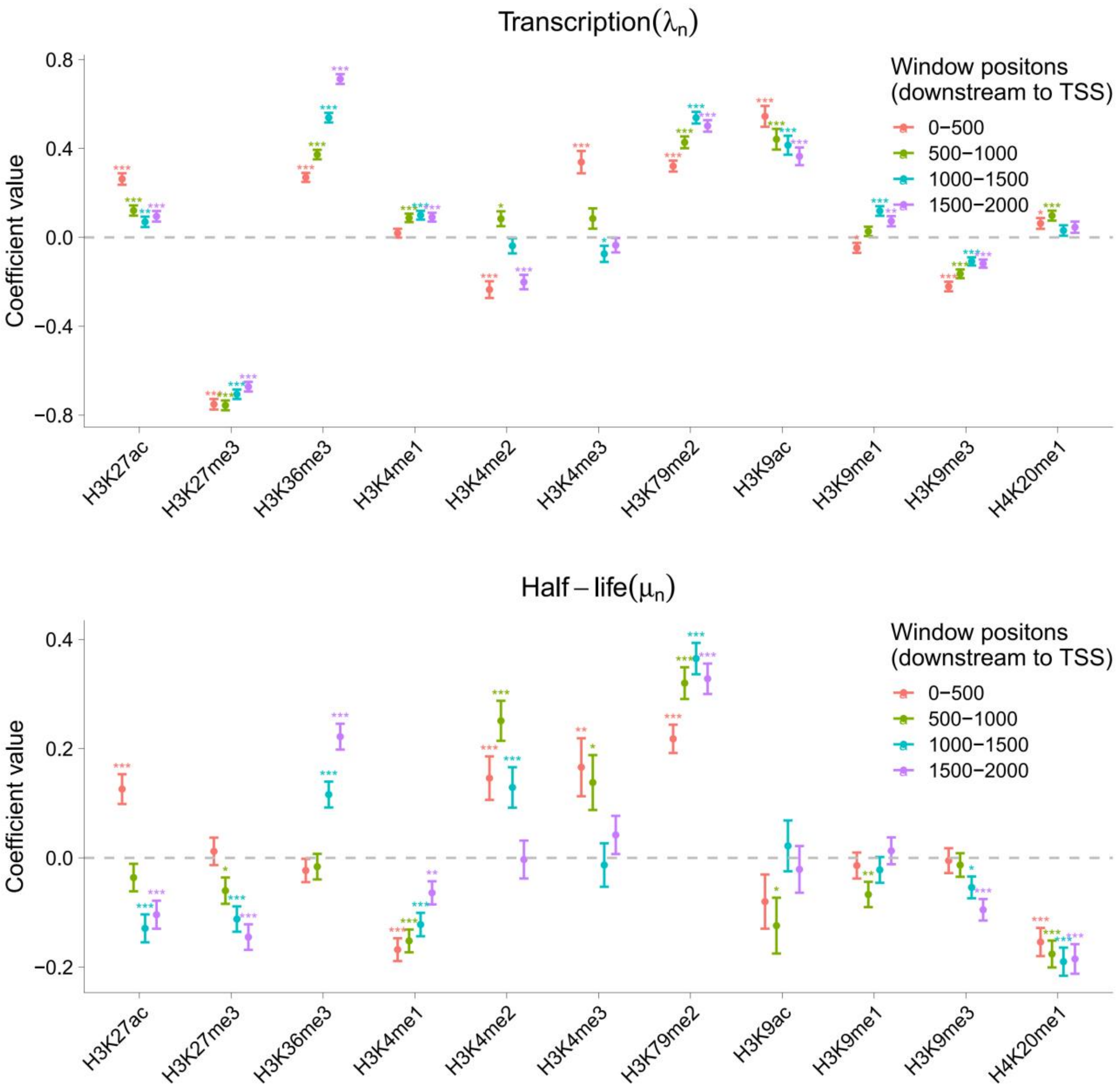

Supplemental Figure 31. Estimated SEM coefficients for transcription (λ_n; top) and half-life (μ_n; bottom) for 11 histone modifications. It's assayed by ChIP-seq in 4 windows, each of 500 bases downstream of the TSS (0-500, 500-1000, 1000-1500, 1500-2000, represented by colors). The entire set of mRNA genes was analyzed. Error bars and significance are as in **Fig. 3B.** The analysis is the same as in **Supplemental Figure 30,** but based on newly collected data for this study.
