# Supplementary material for: Characterizing RNA stability genome-wide through combined analysis of PRO-seq and RNA-seq data
Source: BMC Biol. 2021 Feb 15;19:30. doi: 10.1186/s12915-021-00949-x (PMC7885420; doi:10.1186/s12915-021-00949-x)
Supplement: Supplementary file 3 — Additional file 3. Variation in Elongation Rate is Insufficient to Explain Variation in Half-Life. [file 12915_2021_949_MOESM3_ESM.pdf]

Supplemental Text

Variation in Elongation Rate is Insufficient to Explain Variation in Half-Life

We find that the variation in elongation rate across genes is substantially smaller than the variation in estimated half-lives, indicating that it can account for, at most, a small fraction of the observed variation in half-life. In particular, the elongation rate estimates for the ~1700 genes from Veloso et al. range from 0.0200 to 2.16 times the median value. By contrast, our half-life estimates for the same genes range from 0.594 to 32.4 times the median value. Therefore, the half-life estimates exhibit a dynamic range of a factor of 32.4/0.594≅545, whereas the elongation rate estimates exhibit a dynamic range of a factor of only 2.16/0.0200≅108. Thus, the variation in elongation rate is only about a fifth as large as the variation in estimated half-lives. This five-fold difference holds if we remove the top and bottom 5% from each distribution, to mitigate the influence of outliers.
